# A Novel Netrin-1-Derived Peptide Enhances Protection against Neuronal Death and Mitigates of Intracerebral Hemorrhage in Mice

**DOI:** 10.3390/ijms22094829

**Published:** 2021-05-02

**Authors:** Lin Liu, Kai-Jie Liu, Jian-Bo Cao, Jing Yang, Hua-Li Yu, Xiao-Xiao He, Zi-Xuan He, Xiao-Juan Zhu

**Affiliations:** Institute of Genetics and Cytology, Northeast Normal University, Changchun 130021, China; liul467@nenu.edu.cn (L.L.); liukj249@nenu.edu.cn (K.-J.L.); caojb874@nenu.edu.cn (J.-B.C.); yangj357@nenu.edu.cn (J.Y.); yuhl178@nenu.edu.cn (H.-L.Y.); hexx100@nenu.edu.cn (X.-X.H.)

**Keywords:** intracerebral hemorrhage, hemin, neuroprotection, Netrin-1, peptide

## Abstract

It has been reported that Netrin-1 is involved in neuroprotection following injury to the central nervous system. However, the minimal functional domain of Netrin-1 which can preserve the neuroprotection but avoid the major side effects of Netrin remains elusive. Here, we investigated the neuroprotective effect of a peptide E1 derived from Netrin-1′s EGF3 domain (residues 407–422). We found that it interacts with deleted colorectal carcinoma (DCC) to activate focal adhesion kinase phosphorylation exhibiting neuroprotection. The administration of the peptide E1 was able to improve functional recovery through reduced apoptosis in an experimental murine model of intracerebral hemorrhage (ICH). In summary, we reveal a functional sequence of Netrin-1 that is involved in the recovery process after ICH and identify a candidate peptide for the treatment of ICH.

## 1. Introduction

Intracerebral hemorrhage (ICH) is a devastating stroke subtype with a high mortality and morbidity rate [1,2,3] that involves abnormal rupture of cerebral blood vessels within the brain [4,5]. Surgical removal of hematomas is preferred for achieving hemostasis and relieving intracranial pressure [6]. However, remnant hematic residues and blood breakdown products can still promote secondary brain injury following ICH, inducing neuronal damage and delaying functional recovery [7,8,9]. Thus, the development of effective strategies targeting secondary brain injury induced neuronal damage would be beneficial for stroke treatment.

Netrin-1 is one of the most well-studied proteins that regulate axonal guidance and synaptogenesis [10,11,12] via interaction with its receptors deleted in colorectal cancer (DCC) and UNC5H (unco-ordinated-5 homolog) to activate cell survival, differentiation and proliferation [13,14,15]. The receptors induce a death signal to mediate apoptosis when the Netrin-1 is absent [16]. It is known that the binding of Netrin-1 with DCC results in dimerization of DCC and initiates tyrosine 861 phosphorylation of focal adhesion kinase (FAK) to induce neurite outgrowth and axonal guidance [17,18]. The binding of Netrin-1 with DCC involves the EGF3 domain of Netrin-1 and the FN5 domain of DCC in activation of Netrin-1 signaling pathway [19,20]. Recently, Netrin-1 was shown to exert neuroprotective effects in ischemic regions [21]. The expression level of Netrin-1, which inhibits cell apoptosis and promotes neuronal regeneration, in the serum is positively correlated with patient recovery from ischemic stroke [22,23].

Interestingly, transplantation of bone marrow mesenchymal stem cells that secrete Netrin-1 into the damaged rat sciatic nerve initiates axonal regeneration [24] and reduces motor neuron death [25]. Stereotaxic injection of the Netrin-1 protein into the ventricles reduces nerve cell death after subarachnoid hemorrhage through activation of DCC/APPL-1/AKT signaling [26]. A polypeptide derived from the EGF3 domain of Netrin-1 (amino acids 423–433) markedly activates ERK phosphorylation and promotes the production of NO, which has cardioprotective effects [27,28]. However, Netrin-1 has been identified as a potential biomarker for tumorigenesis [29,30,31] because regulation of the Netrin-1 status is found in multiple tissue-derived cancers [32]. When Netrin-1 is bound to its receptor DCC or Unc5H, the receptors transduce a positive signal leading to survival, inflammation, angiogenesis and anti-apoptosis, which in turn regulates tumorigenesis [31]. Therefore, it is very important to identify the minimal functional domain of Netrin-1 that can preserve the neuroprotection and avoid the major side effects of Netrin-1.

Here, we found that peptide E1 (residues 407–422), which is derived from the EGF3 domain of Netrin-1, interacts with DCC to protect neurons from hemin-induced toxicity. In an experimental model of ICH mice, the application of peptide E1 improved functional recovery, as determined by behavioral assays. Furthermore, peptide E1 can prevent neurons from degenerating after ICH. On the basis of these results, we identified a functional sequence of Netrin-1 that is involved in recovery after ICH and determined that this peptide may be used for the treatment of ICH.

## 2. Results

### 2.1. EGF3 Domain Is Critical for the Netrin-DCC Interaction

**Figure 1:**
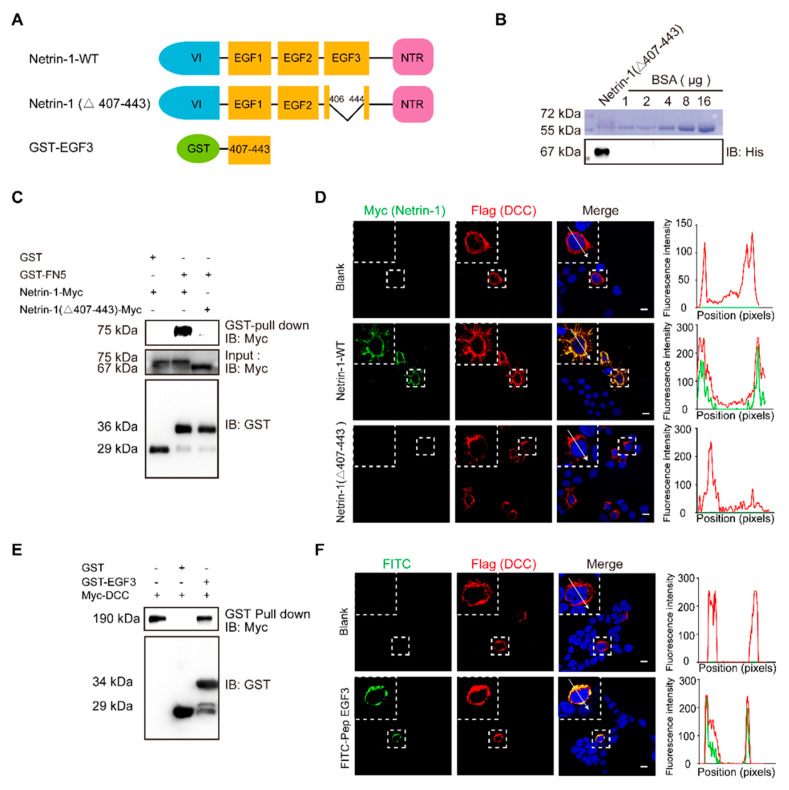
The EGF3 domain of Netrin-1 is the critical region for Netrin-1/DCC interaction. (**A**) Illustrations of Netrin-1 constructs. The Netrin-1 constructs are Netrin-1 full-length, Netrin-1-(∆407–443) and GST-EGF3. (**B**) Purified Netrin-1-(∆407–443)-Myc/His were quantified through a comparison with the BSA standard curve. (**C**) Immunoblotting of the pull-down fraction by the GST-DCC FN5 domain fusion protein and GST alone. (**D**) Immunofluorescence images of Flag-DCC expressing 293T cells incubated with control medium, Netrin-1-myc/his and Netrin-1-(∆407–443) -Myc/his for 30 min, respectively, and the quantitative analysis. Scale bar, 10 µm. (**E**) Immunoblotting of the pull-down fraction by the GST-EGF3 fusion protein and GST along. (**F**) Immunofluorescence images of Flag-DCC expressing 293T cells incubated with control medium and FITC labeled Pep EGF3 for 30 min, respectively, and the quantitative analysis. Scale bar, 10 µm.

Netrin-1 consists of a VI domain, three EGF repeats, and a *C*-terminal domain [33]. The crystal structure of the human Netrin-1/DCC complex reveals that Netrin-1 EGF3 domain is crucial for the Netrin-1/DCC interaction, but not the EGF1 and EGF2 domains [34]. Mutagenesis key amino acid residues (His407, Gln442 or Gln443) at EGF3 domain completely disrupted Netrin-1/DCC binding [19]. EGF1/2 domains are required for switching attractive signaling into repulsive signaling when Unc5 coexists with DCC [20]. Thus, we first generated full-length and EGF3-deleted (Netrin-1 Δ407–443) Netrin-1 constructs (Figure 1A) and transiently expressed these constructs in cultured HEK293T cells for GST pulldown. Binding was observed for the constructs that expressed Netrin-1 but not Netrin-1 (Δ407–443) (Figure 1C). These results suggest that the domain of EGF3 containing amino acids 407–443 is responsible for the interaction of Netrin-1 with DCC.

To investigate the role of EGF3 domain in Netrin-DCC, we first purified myc-tagged Netrin-1 or truncated Netrin-1 (Δ407–443) (Figure 1B). Next, HEK293T cells were transfected with a plasmid carrying DCC cDNA and incubated with Netrin-1 protein, and then Netrin-1 binding was assessed by immunostaining. We found that full-length Netrin-1, but not truncated Netrin-1, was able to bind to DCC at the plasma membrane (Figure 1D). To determine whether the EGF3 domain can interact with DCC, we generated a GST-EGF3 construct to pull down lysed cells overexpressing DCC. We observed specific binding of DCC with GST-EGF3 (Figure 1E). Furthermore, we synthesized the FITC-labelled peptide EGF3 and confirmed that, like full-length Netrin-1, it was able to bind to DCC at the plasma membrane (Figure 1F). Taken together, our data indicate that the EGF3 domain is responsible for the interaction of Netrin-1 with DCC.

### 2.2. The EGF3 Domain Induces Phosphorylation of Downstream Pathways of Netrin-1

**Figure 2:**
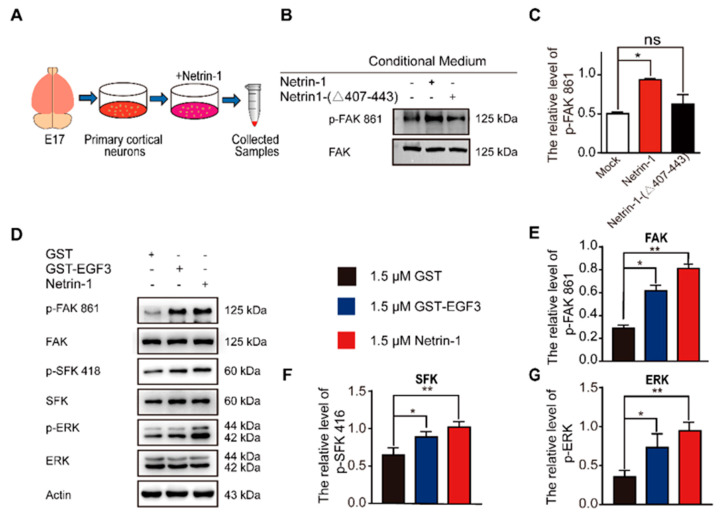
EGF3 domain activates downstream pathways of Netrin-1. (**A**) Experimental procedure. (**B**) Cortical neurons (E17 and DIV3) were stimulated with Netrin-1, Netrin-1-(∆407–443) or control medium for 20 min. Cell lysates were incubated with an anti-p-FAK-861 antibody to confirm Netrin-1 activity. (**C**) Quantitative analysis of the western blot results shown in (**B**). *n* = 3. (**D**) Cortical neurons (DIV3) were treated with 1.5 μM GST (+), 1.5 μM GST-EGF3 fusion protein (+) or 1.5 μM Netrin-1 (+) medium for 20 min. Cell lysates were incubated with anti-p-FAK-861, anti-p-SFK-418 and anti-p-ERK-202/204. (**E**–**G**) Quantitative analysis of the western blot results shown in (**D**). *n* = 3. Data are presented as the means ± SEM. One-way ANOVA was used for all statistical analyses shown in this figure (* *p* < 0.05, ** *p* < 0.01; ns, not significant). Details of data analysis are seen in Supplementary Table S1.

The interaction of Netrin-1 and DCC activates multiple signaling pathways, including FAK, SFK and ERK [16,35]. Focal adhesion kinase (FAK) is one of the major tyrosine phosphorylation activities linked to Netrin-1/DCC signaling. Netrin-1 activates FAK phosphorylation and Src family protein tyrosine kinases (SFKs) phosphorylation [17]. Both FAK and SFK signaling pathways play a pivotal role in the developmental stage of nervous system and injury repair [36,37]. Moreover, Netrin-1 is able to activate the extracellular signal-regulated kinase-1/2 (ERK1/2), which mediates action of Mitogen-activated protein kinase (MAPK) pathway [38,39,40]. To investigate whether the EGF3 domain is critical for the activation of downstream signaling pathways via the binding of Netrin-1 to DCC, we first examined the effect of the EGF3 domain on the tyrosine 861 phosphorylation of FAK (Figure 2A). We found that deletion of EGF3 inhibited FAK phosphorylation in cultured cortical neurons (Figure 2B,C), thus confirming an essential role for EGF3 in the Netrin-1 signaling pathway. Next, cultured cortical neurons were stimulated with the GST-EGF3 fusion protein for 20 min, and western blotting further confirmed that treatment with the GST-EGF3 fusion protein induced FAK, ERK and SFK phosphorylation (Figure 2D–G). Taken together, these results suggest that the EGF3 domain of Netrin-1 functions as a potent stimulator to activate downstream signaling pathways.

### 2.3. Peptide E1 and E2 Interact with DCC to Activate the Downstream Signaling Pathway of Netrin-1

**Figure 3:**
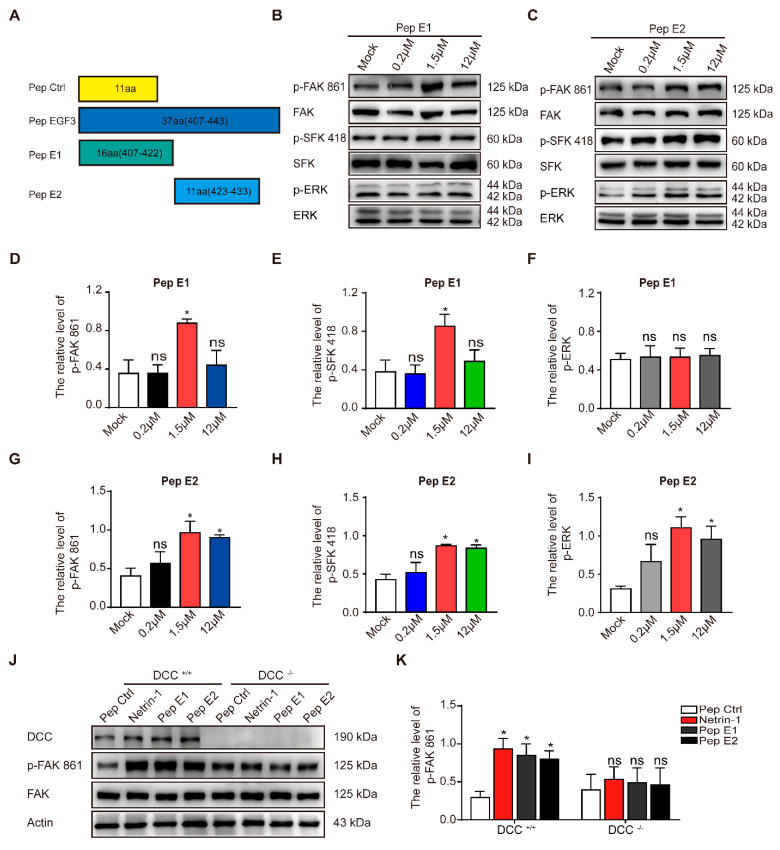
The minimal sequences of the EGF3 domain activates downstream pathways of Netrin-1. (**A**) Illustrations of the peptides. (**B**) Cortical neurons (DIV3) were stimulated with gradient concentrations of peptide E1. Cell lysates were incubated with anti-p-FAK-861, anti-p-SFK-418 and anti-p-ERK-202/204. (**C**) Cultured cortical neurons were treated with gradient concentrations of peptide E2. Lysates were collected and incubated with the indicated antibodies to measure the phosphorylation of FAK, SFK and ERK. (**D**–**F**) Quantification of the extent of FAK, SFK and ERK phosphorylation induced by peptide E1 in neurons is shown in (**B**). *n* = 3. (**G**–**I**) Quantification of the extent of FAK, SFK and ERK phosphorylation induced by peptide E2 is shown in (**C**). *n* = 3. (**J**) Cortical neurons derived from DCC wild-type and homozygote mutant embryos were stimulated with Netrin-1 or Netrin-1-derived peptides and were lysed. The resulting lysates were subjected to immunoblotting. (**K**) Quantification of FAK PY861 and DCC levels in DCC mutant neurons is shown in (**J**). *n* = 3. The data are presented as the means ± SEMs. One-way ANOVA was used for all statistical analyses shown in this figure (* *p* < 0.05; ns, not significant). Details of data analysis are seen in Supplementary Table S1.

As previously described, a small peptide derived from the EGF3 domain of Netrin-1 (amino acids 423–433) induced ERK phosphorylation [27]. We synthesized peptides E1 (residues 407–422) and E2 (residues 423–433) that were derived from the EGF3 domain of Netrin-1 (Figure 3A). We found that incubation of peptide E1 increased the levels of phosphorylated FAK and SFK (Figure 3B,D,E) in the culture cells in a dose-dependent manner but did not alter the levels of phosphorylated ERK (Figure 3B,F). Unlike peptide E1, peptide E2 not only induced the tyrosine phosphorylation of FAK (Figure 3C,G) and the tyrosine phosphorylation of SFK (Figure 3C,H) but also induced the tyrosine phosphorylation of ERK (Figure 3C,I) in cultured cortical neurons. The control peptide (Pep Ctrl) had no detectable effect on the tyrosine phosphorylation of FAK, SFK and ERK (Figure S1). Furthermore, we observed that the phosphorylation of components of the FAK signaling pathway induced by peptide E1 was time-dependent (Figure S2). These results indicate that E1 and E2 are the minimal functional peptides of Netrin-1.

To determine whether the activation of the FAK signaling pathway is DCC-dependent, we measured the extent of tyrosine phosphorylation of FAK induced by these peptides in DCC^+/+^ and DCC^−/−^ neurons. We found that both peptides E1 and E2 failed to induce the phosphorylation of FAK in DCC^−/−^ neurons (Figure 3J,K). We also found that synthesized N-terminally Flag-tagged peptide E1 and E2 (Flag-E1 and Flag-E2) direct bind with DCC-FN5 domain (Figure S3).

### 2.4. Peptide E1 Protects Neurons from Hemin-Induced Cell Death In Vitro

**Figure 4:**
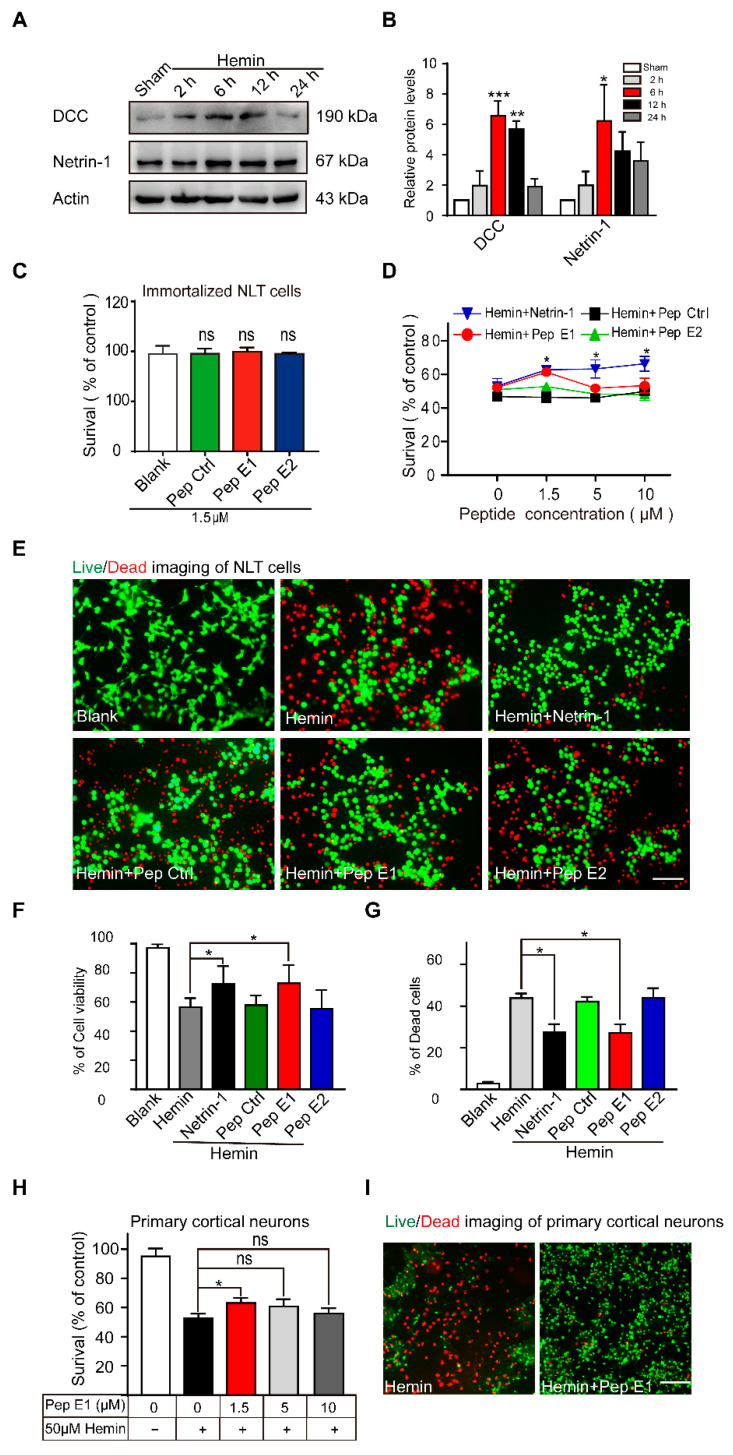
Peptide E1 ameliorates hemin induced cell death in vitro. (**A**) Western blot of Netrin-1 and DCC in cultured NLT cells exposed to Hemin (60 µM). (**B**) Quantitative analysis of the western blot results shown in (**A**). *n* = 3. (**C**) CCK-8 assays showed that series peptides were not changed cell viability. *n* = 4. (**D**) 1.5 µM Pep E1 preserved NLT cell viability when added well after exposure to hemin (60 µM). *n* = 4. (**E**) Live (green)/Dead (red) assay of NLT cells exposed to hemin (60 µM, 6 h). Scale bar, 50 μm. (**F**,**G**) Quantitative analysis of the live/dead assay in (**E**). *n* = 9. (**H**) CCK-8 assays showed that Pep E1 could reduce hemin induced cell death in primary culture neurons. *n* = 8. (**I**) Live/Dead assays imaging of primary culture neurons (50 µM, 6 h). Scale bar, 50 μm. Data are presented as the means ± SEM. One-way ANOVA or Two-way ANOVA was used for all statistical analyses shown in this figure (* *p* < 0.05, ** *p* < 0.01, *** *p* < 0.001; ns, not significant). Details of data analysis are seen in Supplementary Table S1.

To exhibit the protection against neuronal injure of peptide E1, we employed an Hemin inducible toxicity reactions to mimic secondary brain injury after intracerebral hemorrhage [41,42]. Cultured GnRH-expressing neuronal cells (NLT) [43,44,45] were incubated with hemin at different concentrations and durations. Incubation of NLT cells with hemin at the medial lethal dose (60 µM) resulted in a time-dependent decrease in the cell survival rate, which suggests that hemin was taken up by neurons and metabolized (Figure S4). To examine whether the expression of Netrin-1 and DCC responded to the hemin-induced neuronal cell death, cultured NLT cells were incubated with hemin for different durations. The western blot results demonstrated that the expression levels of Netrin-1 and DCC were increased significantly at 6 h after hemin incubation and then declined gradually (Figure 4A,B). To investigate whether peptides E1 and E2 were able to attenuate hemin-induced neuronal death, we performed a CCK-8 assay with Netrin-1, Pep Ctrl, peptide E1 and peptide E2. We found that these peptides had no detectable effect on cell viability (Figure 4C and Figure S5). Compared to incubation with Pep Ctrl and peptide E2, peptide E1 and Netrin-1 ameliorated neuronal death induced by hemin (Figure 4D). We also stained with calcein-AM and PI to distinguish live and dead cells. The results showed that both Netrin-1 and peptide E1 protected against hemin-induced death in vitro (Figure 4E–G). Furthermore, 1.5 μM peptide E1 alleviated hemin-induced death in primary cultured neurons (Figure 4H,I). Altogether, these results showed that peptide E1 protects neurons from hemin-induced cell death.

### 2.5. Netrin-1-Derived Peptide E1 Promotes Functional Recovery after ICH

To investigate whether peptide E1 derived from Netrin-1 play an important role in functional recovery after hemorrhage stroke, we established an experimental ICH model in mice by injecting collagenase mixed with ink into the striatum (Figure S6A). After three days, images of brain slice were shown to trackers the inject position and collagenase volume with ink (Figure S6B,C). Mice with striatal hemorrhage exhibited weakness on the ipsilateral side because of brain injury (Figure S7A–C). Hematoma in the striatum following ICH were visible compared with injection of saline (Figure S7D). Obviously, the relative protein levels of Netrin-1 and DCC in the perihematomal cortex were increased in ICH mice compared to control mice on day 2 and the Netrin-1 was still higher significantly at day 3, indicating that Netrin-1 is important for recovery of brain injury (Figure S7E,F).

**Figure 5:**
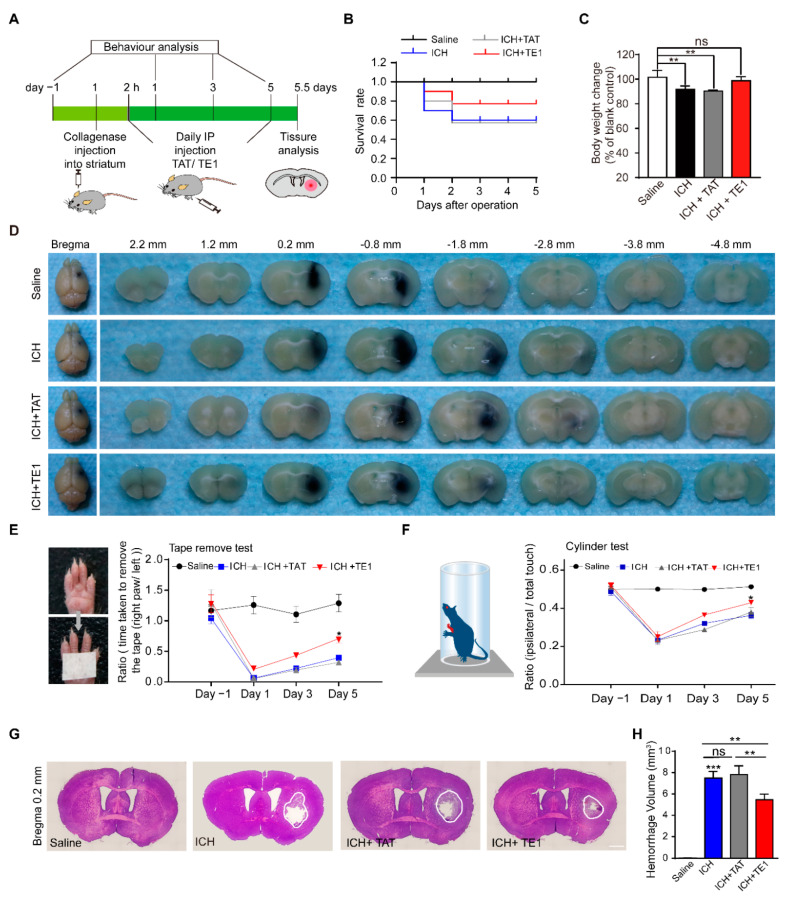
Netrin-1 derived peptide E1 promotes recovery process after ICH. (**A**) Schematic of the experimental paradigm of intraperitoneal (IP) injection of peptide TE1 in ICH modeling of mice. (**B**) Survival ratio post-ICH of each group was recorded, the Kaplan-Meier method was employed. *n* = 10. (**C**) Body weight changes at 5 days after ICH. *n* = 6. (**D**) Collage/Saline mix with equal ratio of ink (total 0.6 µL) injected in striatum of C57 mice, representative images showing ink area were similar in mice treat with/without peptide TE1 5 days after ICH. (**E**,**F**) IP injection of peptide TE1 (12 mg/kg) but not TAT (12 mg/kg) control, improved behavior as monitored by a tape removal task (**E**) or a corner task (**F**). Saline, *n* = 10 individual animals; ICH, *n* = 6 individual animals; ICH + TAT, *n* = 6 individual animals; ICH + TE1, *n* = 8 individual animals. (**G**) Representative hematoxylin and eosin-stained sections in mice treat with/without peptide TE1 5 days after ICH. Scale bar, 1 mm. (**H**) Quantitation of hemorrhage volume in brain sections shown in (**G**). Saline, *n* = 10 individual animals; ICH, *n* = 6 individual animals; ICH + TAT, *n* = 6 individual animals; ICH + TE1, *n* = 8 individual animals. Data are presented as the means ± SEM. One-way ANOVA or Two-way ANOVA was used for all statistical analyses shown in this figure (* *p* < 0.05, ** *p* < 0.01, *** *p* < 0.001; ns, not significant). Details of data analysis are seen in Supplementary Table S1.

Next, FITC-labelled TAT-peptide E1 (FITC-Pep TE1), which added a TAT sequence of the *N*-terminus of peptide E1, was injection to the ICH mice and the FITC-Pep TE1 was shown to cross blood-brain barrier (Figure S8). The ICH and control mice were intraperitoneally injected with the peptide (12 mg/kg) daily or an equal volume of saline (Figure 5A). We found that administration with peptide TE1 improved the survival rate and rescued defects in body weight after ICH (Figure 5B,C). We also found collagenase volume traced with equal ratio of ink in each group was similar, confirmed this standard ICH model of collagenase resulted in reproducible intrastriatal hematomas (Figure 5D). Obviously, peptide TE1 improved the behavioral scores of the ICH mice in the tape removal test (Figure 5E) and cylinder test (Figure 5F). As expected, peptide TE1 reduced the infarct volume after ICH (Figure 5G,H). Taken together, these results suggest that peptide E1 was able to reduce the hematoma volume and improve functional recovery in a mouse model of ICH.

### 2.6. Netrin-1-Derived Peptide E1 Reduces Neuronal Apoptosis after ICH

**Figure 6:**
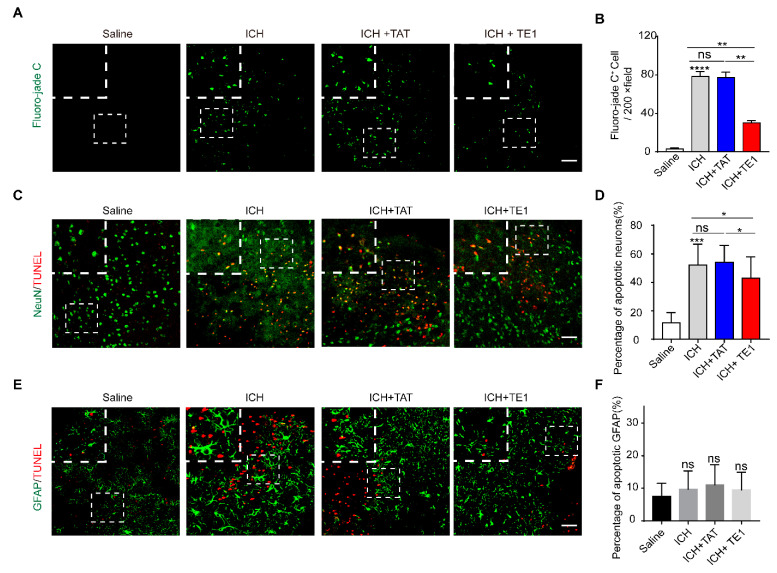
Netrin-1 derived peptide E1 can protect against neuronal death and degeneration, but not astrocytes after ICH. (**A**) Representative Fluoro-jade C-stained sections in mice treat with/without TE1 at 5 days after ICH. Scale bar, 50 μm and 10 μm, respectively. (**B**) Quantitation of Fluoro-jade C positive cells in the perihematomal region (*n* = 4). (**C**) TUNEL staining showing effects of TE1 on secondary brain injury (SBI) at 5 days after ICH onsets. Representative images from Saline, ICH, ICH + TAT, ICH + TE1 groups. Each group was subjected to ICH except for the Saline group. Scale bar, 50 μm and 10 μm respectively. (**D**) Quantitative analyses for the percentage of TUNEL-positive neurons shown in (**C**). *n* = 4. (**E**) Colocalization of TUNEL with GFAP at 5 days after ICH. Representative images from Saline, ICH, ICH + TAT, ICH + TE1 groups. Scale bar, 50 μm and 10 μm, respectively. (**F**) Quantitative analyses for the percentage of TUNEL-positive astrocytes shown in (**E**). *n* = 4. Data are presented as the means ± SEM. One-way ANOVA was used for all statistical analyses shown in this figure (* *p* < 0.05, ** *p* < 0.01, *** *p* < 0.001, **** *p* < 0.0001; ns, not significant). Details of data analysis are seen in Supplementary Table S1.

To explore the underlying mechanisms of peptide E1 in the process of functional recovery of ICH. We detected neuronal degeneration in the perihematomal region by fluoro-jade C (FJC) staining. We found that the administration of peptide TE1 significantly reduced the number of FJC-positive cells in perihematoma regions, indicating that pep TE1 inhibited neuronal degeneration after ICH (Figure 6A,B). We performed TUNEL staining and found that peptide TE1 significantly decreased the number of TUNEL-positive neurons (Figure 6C,D). The number of TUNEL-positive astrocytes was no detectable change after peptide TE1 treatment (Figure 6E,F). In addition, by using FITC-labelled 4 kDa dextran as a tracer to indicate permeability of blood–brain barrier (BBB), we found that peptide TE1 had no effect on the recovery of BBB permeability (Figure S9A,B). The serum raw fluorescence units (RFUs) levels were similar, confirmed the presence of injected tracer in all the animals analyzed (Figure S9C). We also used fluorescein-labelled lectin to label microvessels but failed to observe any improvement after TE1 treatment (Figure S9D,E). Above all, these findings suggest that the peptide E1 promotes neuronal recovery after ICH by inhibiting neuronal apoptosis.

## 3. Discussion

Our study identified a core peptide sequence of Netrin-1 that is crucial to functional recovery after ICH. By interacting with DCC, this Netrin-1-derived peptide protected against neuronal death due to secondary damage after ICH and is thus a promising treatment for ICH.

The therapeutic application of Netrin-1, a high-molecular-weight protein, requires identification of the minimal functional sequence of Netrin-1 that promotes downstream signaling. In our study, peptides E1 (residues 407–422) and E2 (residues 423–433), which are derived from the EGF3 domain of Netrin-1, respectively, were generated. We found that peptide E1 (residues 407–422) protected against neuronal death after ICH.

We found that Netrin-1 and DCC expression levels were increased in NLT cells after hemin treatment. Hemin is released from hemoglobin after ICH and induces secondary injury to both glia and neuronal cells [46,47,48]. Additionally, we also observed increased protein levels of Netrin-1 and DCC following experimental ICH. Additionally, we also observed increased protein levels of netrin-1 and DCC following experimental ICH. These results are consistent with previous results showing that netrin-1 plays an important role in recovery after ICH [49].

Furthermore, we found that peptide E1 specifically activated the FAK and SFK signaling pathways. Therefore, peptide E1 may have highly specific effects and be effective in the recovery process after ICH. Unlike peptide E1, peptide E2 can activate ERK signaling, which promotes neuronal death. This may be caused by a Cx(1–2)Cx(3–4)Tx(0 –1)G motif in E2 which is able to activate ERK signaling pathway [28]. This indicates that different Netrin-1-derived peptides perform different functions.

The main receptors of Netrin-1 are DCC and members of the UNC5 family [50]. The role of DCC and UNC5H2 in ICH remains controversial [51,52]. The sequence of Netrin-1 we identified is critical to the interaction of Netrin-1 with DCC [19]. These results suggest that DCC may participate in Netrin-1-induced functional recovery after ICH. Further studies are needed to investigate the underlying mechanisms of these processes and the related signaling pathways. In future experiments, we will assess the biological properties of peptide E1 to explore its toxicity and half-life for clinical application.

## 4. Materials and Methods

### 4.1. Animals

The laboratory was a specific pathogen-free (SPF) laboratory. All mice were housed under a 12-h light-dark cycle with lights on from 6 am to 6 pm and provided access to food ad libitum. DCC^+/−^ mice were generated as previously described [53]. Embryos of DCC^+/−^ mice were used for cortical neuronal culture. Genotyping was carried out by polymerase chain reaction (PCR) as described previously [53]. C57BL/6J mice were purchased from Beijing Huafukang Bioscience Co., Ltd. (Beijing, China).

### 4.2. Constructs

Expression constructs encoding human DCC (HGNC: 2701), human Netrin-1 (HGNC: 8029) and human UNC5A (HGNC:12567) were generated in our laboratory [14,32,45]. pcDNA 3.1-hNetrin-1 (∆407–443)-myc-His was generated with a Seamless Assembly Cloning Kit (Clone Smarter, C5891, USA). cDNA sequence of hNetrin-1 (∆407–443) was seen in Supplementary Table S2. cDNA sequences corresponding to the FN5 domain of DCC and amino acids 407–443 of Netrin-1 were constructed by ligation into the pGEX-5x-1 vector to produce GST fusion proteins. 

### 4.3. Collection of Netrin-1 Conditioned Medium

Netrin-1-conditioned medium was collected and validated as described in our previous report [13,14]. Briefly, Myc-His-tagged human Netrin-1 was expressed in HEK293T cells. After 24 h, the cells were washed 3 times with Opti-MEM (Invitrogen, 31985070, Carlsbad, CA, USA) and then incubated for three days in serum-free Opti-MEM. The media was enriched by centrifugal filtration (Millipore, UFC903096, Billerica, MA, USA) and stored at −80 °C. Enriched Netrin-1 was immunoblotted with anti-His and quantified by comparison with BSA as a loading control. The effect of Netrin-1 was then assessed by phosphorylation of FAK. Normally, 0.1 mg/mL Netrin-1 effectively induced FAK phosphorylation, and this concentration was used in our studies. The duration of Netrin-1 stimulation was 20 min in all experiments unless indicated otherwise.

### 4.4. Western Blot Analysis

Western blotting was performed as previously described [54]. The samples were lysed in lysis buffer (1% Nonidet P-40, 0.5% sodium deoxycholate, 0.1% SDS, 1 mM EDTA, 1 mM EGTA, 150 mM NaCl, 10% glycerol, 1% Triton X-100, 100 mM NaF, 1 mM vanadate, and protease inhibitors) to lyse cells and fresh brain tissue. The primary antibodies used were: mouse anti-DCC (1:500, #554223, BD, San Jose, CA, USA ), mouse anti-His (1:1000, TA-02, ZSGB-BIO, Beijing, China), mouse anti-myc (1:1000, 22E8, Sungene Biotech, Tianjin, China), mouse anti-Netrin-1 (1:1000, ALX804838, Enzo Life Sciences, Farmingdale, New York, USA ), mouse anti-flag (1:1000, F3165, Sigma Aldrich, St Louis, MO, USA), rabbit anti-phospho-FAK(pTyr861) (1:1000, F9176, Sigma Aldrich, St Louis, MO, USA), rabbit anti-FAK (1:200, sc-557, Santa Cruz, Santa Cruz, CA, USA), rabbit anti-phospho-SRC (Tyr418)(1:1000, Invitrogen, Carlsbad, CA, USA), rabbit anti-SRC Family (1:1000, #2123, CST, Danvers, MA, USA), mouse anti-GAPDH (1:5000, TransGene Biotech, Beijing, China), mouse anti-beta actin (1:3000, A5441, Sigma Aldrich, St Louis, MO, USA), rabbit anti-phospho-ERK(Thr202/Tyr204) (1:1000, #9101, CST, Danvers, MA, USA), rabbit anti-ERK (1:1000, #4695, CST, Danvers, MA, USA), HRP-conjugated secondary antibody against mouse, goat or rabbit were purchased from Jackson ImmunoResearch (West Grove, PA, USA).

### 4.5. Cell Surface Binding

HEK293 cells plated on coverslips were transfected with Flag-DCC plasmids using the calcium phosphate method as described previously [55]. Approximately 40 h after transfection, the cells were incubated with 100 μg/mL myc-Netrin-1 or myc-Netrin-1 (∆407–443) for 30 min before being washed for 5 min with HBHA (20 mM HEPES, pH 7.0, and 0.5 mg/mL BSA in HBSS), rinsed with 1× PBS, and fixed sequentially with 4% paraformaldehyde in PBS for 10 min. The primary antibodies that were used were rabbit anti-myc (1:200, Abcam, ab9106, Cambridge, MA, USA) and mouse anti-flag (1:100, Sigma Aldrich, F3165) antibodies. The secondary antibodies that were used were Alexa Fluor 488-conjugated donkey anti-rabbit (1:1000; Invitrogen, A21206) and Alexa Fluor 546-conjugated goat anti-mouse (1:1000; Invitrogen, A10036) antibodies. The nuclei were stained with 4′,6-diamino-2-phenylindole (DAPI) (Thermo Fisher, Waltham, MA, USA). All images were acquired with a Zeiss LSM 880 confocal microscope.

### 4.6. GST Pull-Down

The GST-DCC fusion protein was purified with glutathione-SepharoseTM 4B beads (GE Healthcare, Little Chalfont, Buckinghamshire, UK) in accordance with the manufacturer’s protocol. Approximately 500 μg of cell lysate from HEK293T cells transfected with the indicated plasmids was incubated with 2–5 μg of GST-DCC-FN5 fusion protein in RIPA buffer at 4 °C overnight. Glutathi-one-SepharoseTM 4B beads were used to capture the GST-DCC-FN5 fusion protein and its interacting proteins. The bound proteins were released into protein loading buffer by heat denaturation.

### 4.7. Primary Cortical Neuron Culture

Primary cortical neurons were cultured as previously described [56]. In brief, embryos (E17) were removed from anaesthetized pregnant mice. The cerebral cortices were separated and chopped into small pieces. After incubation in 0.125% Trypsin Plus with 0.05% DNase in HBSS at 37 °C for 20 min, the cells were triturated with fire-polished glass Pasteur pipettes and filtered with a 40 µm filter. Dissociated cells were suspended in DMEM with 10% FBS and plated on poly-d-lysine-coated dishes or glass coverslips at 37 °C in a 5% CO_2_ atmosphere. After 4 h, the medium was replaced with neurobasal medium supplemented with B27 and GlutaMAX.

### 4.8. NLT Cell Culture and Treatment Protocols

Mouse GnRH-expressing neuronal cells (NLT) was maintained by our laboratory. NLT cells were grown in DMEM (Sigma Aldrich, St Louis, MO, USA) contain 10% FBS (Biological Industries, Kibbutz Beit Haemek, Israel) and 1% penicillin/streptomycin (Thermo Fisher Scientific, Waltham, MA, USA) at 37 °C under a humidified atmosphere of 95% air and 5% CO_2_. To determine the neurotoxicity of hemin, various concentrations (0, 10, 30, 60, 90 and 120 µM) of hemin (Sigma-Aldrich) were added to NLT cells and treated for 6 h. Of these concentrations, 60 µM (LD50) was selected and used in the subsequent experiments. Control groups were treated with vehicle (DMSO) only. Cell viability and Live/Dead staining were performed 6 h after treatment. Three wells were used for each group. Experiments were repeated at least 3 times.

### 4.9. In Vitro Model of Hemin-Induced Cell Death

Cell death was induced in primary cortical neurons and NLT cells by treatment with 50μM and 60 μM hemin, respectively, as previously described [57,58]. Hemin was dissolved in 0.1 M NaOH to generate a stock solution. For the neuroprotection studies, cells were treated with hemin 6h in the presence of designated agents or sodium selenite dissolved in double distilled water.

### 4.10. CCK-8 Assay

Cell viability was measured by the CCK-8 assay (CK04, Dojindo, Kumamoto, Japan) as previously described [59]. In brief, Neuronal were seeded in 96-well plates at a density of 6000 cells/well in 100 μL culture medium and fed in the incubator overnight. Cells were treated with Hemin for 6 h. The neuronal cells were washed in prewarmed PBS (37 °C) and added CCK-8 reagent (10 μL per 100 μL medium) to each well. After that, we incubated the plates for 2 h in a 5% CO_2_ atmosphere, and the absorbance at 450 nm was measured using a microplate reader. Cell viability was represented as percent of the control (untreated cells).

### 4.11. Live/Dead Staining

The ability of the CCK-8 assay to measure viability was verified by staining with calcein-AM/PI in PBS (Live/Dead Assay, KeyGEN BioTECH, Nanjing, China) according to the manufacturer’s instructions. The staining solution consisted of 2 μM calcein AM reagent and 8 μM PI reagent mixed in PBS. Samples were incubated for 30 min and imaged using a 20x objective lens of a fluorescence microscope (Olympus FSX100, Tokyo, Japan). A minimum of 3 independent cell cultures were used for the in vitro model of hemin-induced cell death.

### 4.12. Peptide Synthesis and Administration

The Pep Ctrl (NH2-TPCCGKTDVGI-CONH2), Pep E1 (NH2- HPVGAAGKTCNQTTGQ -CONH2), Pep E2 (NH2- CPCKDGVTGIT-CONH2), Tat (NH2-YGRKKRRQRRR-CONH2), TE1 (NH2- YGRK-KRRQRRR-HPVGAAGKTCNQTTGQ-CONH2), FITC labelled TE1 (NH2- YGRK-KRRQRRR-HPVGAAGKTCNQTTGQ-CONH2), FITC labelled Pep EGF3 (NH2-HPVGAAGKTCNQTTGQCPCKDGVTGITCNRCAKGYQQ-CONH2), Flag labelled Pep Ctrl (NH2-DYKDHDGDYKDHDIDYKDDDDK-GGGGS-TPCRCGKTYDVGI-CONH2), Flag labelled Pep E1 (NH2- DYKDHDGDYKDHDIDYKDDDDK-GGGGS-HPVGAAGKTCNQTTGQ -CONH2) and Flag labelled Pep E2 (NH2- DYKDHDGDYKDHDIDYKDDDDK-GGGGS-CPCKDGVTGIT-CONH2) peptides with 99% purity were purchased by Shanghai GL Biochem (Shanghai, China) company and Wuhan Bioyeargene Biosciences (Wuhan, China) company.

Treatment groups of saline, TAT or TE1 (dissolved in saline) were given to mice 2 h after ICH and every day for 5 days by intraperitoneal injection at a total volume of 0.1 mL per animal. Peptides were given in a blinded manner at doses of 12 mg/kg.

### 4.13. Collagenase-Induced Mouse Model of ICH

Collagenase injection is commonly used to induce ICH [60]. Male C57BL/6 mice (8 to 12 weeks of age) were anaesthetized with isoflurane (2 to 5%) and placed on a stereotaxic frame. During the procedure, the body temperature of each animal was maintained at 37 °C with a homeothermic blanket. Using a stereotaxic instrument (Ruiwode Life Science Co., Ltd., Shenzhen, China) and a syringe (Hamilton Company, Reno, NV, USA), 0.3 μL of collagenase (0.045 IU; Sigma-Aldrich, St Louis, MO, USA) was infused into the right striatum (AP: +0.2 mm; ML: −2.3 mm; DV: −3.5 mm) at a flow rate of 0.1 μL/min. A total of 0.3 μL of saline was infused into control animals. All surgical procedures were conducted under aseptic conditions. In this study, over 66 male mice were used, and the animals were randomly assigned to groups before each experiment [57].

### 4.14. Behavioural Analysis

The neurological behavioral analysis of mice after brain hematoma formation was detected by the adhesive tape removal test [61] and cylinder test [60]. The adhesive tape removal test was performed by placing adhesive tape on the planter region of each forepaw (right and left) of the mice. Each mouse was placed in a novel cage. The time from when the tape was applied to when the mouse successfully removed it was recorded for each paw. A maximum of 300 s was allowed for each paw. Between experiments, the animals were housed in their home cages in a pathogen-free facility.

The cylinder test was performed by placing a mouse in a transparent acrylic glass cylinder (diameter: 8 cm; height: 25 cm) in front of 2 mirrors and a camera for 5 min. The camera was placed centrally in front of the 2 mirrors and the cylinder to obtain an optimal video. For assessment of independent forelimb use, (1) the ability of the mice to contact the cylinder wall with one forelimb during full rearing and (2) the ability of the mice to land with only one forelimb on the floor after full rearing were evaluated. At least 20 contact points for each forelimb were counted using slow motion. For baseline analysis before surgery, the test was performed twice per mouse with a 1 h break between trials. Forelimb use is expressed as the ratio of right- to left-sided independent forelimb use.

### 4.15. Haematoma Volume Analysis

To determine if the treatments reduced the hematoma volume, mice were sacrificed 5 days after ICH; the brains were removed and flash frozen in OCT. Coronal sections were sliced at a thickness of 30 µm and placed directly on glass slides. To quantify the hematoma volume, the sections were digitized at standardized coronal levels. A blinded user measured the contours and hematoma in the left and right hemispheres in each section. The hematoma volume and swelling were measured and calculated using ImageJ (NIH). At least 5 brains per group were subjected to hematoma measurements, and the hematoma volume was measured throughout the brain.

### 4.16. Fluoro-Jade C Staining

Neurodegeneration in mice following collagenase-induced ICH was assessed by staining with a Fluoro-jade C Ready-to-Dilute Staining Kit (TR-100-FJT, Biosensis, Thebarton, Australia) according to the manufacturer’s protocol [62]. Briefly, control mice and ICH mice (±i.p. peptide) were sacrificed 5 days after modelling, and the brains were processed according to the protocol used for the immunocytochemistry experiment. Floating brain sections were immersed in 1% sodium hydroxide solution for 5 min, rinsed for 2 min in 70% ethanol, and then rinsed for 2 min in distilled water. Next, the floating sections were immersed in a 0.06% potassium permanganate solution (KMnO4 in distilled water) for 10 min. The sections were washed with ddH_2_O before being immersed in 0.0001% Fluoro-jade staining solution (Fluoro-jade C in distilled water) for 20 min with gentle shaking in the dark. The sections were washed with ddH_2_O, mounted on coated slides and dried at room temperature overnight in the dark. Fluoro-Jade C staining within the perihematomal region was examined using a Zeiss LSM 880 confocal microscope. Quantitation of Fluoro-jade C staining was performed using ImageJ software.

### 4.17. Tissue Immunofluorescence

Tissue immunofluorescence staining was performed using a series of 30-μm-thick sections. Sections were incubated with the following primary antibodies: mouse anti-NeuN (1:500, Millipore, MAB377, Billerica, MA, USA) or mouse anti-GFAP (1:200, Sigma Aldrich, G3893, St Louis, MO, USA) antibodies at 4 °C overnight. After washing with PBS, sections were then incubated with Alexa Fluor 488-conjugated donkey anti-mouse (1:1000; Invitrogen, A21202, Carlsbad, CA, USA) antibody.

### 4.18. TUNEL Assay

A terminal deoxynucleotidyl transferase-mediated deoxyuridine triphosphate (dUTP) nick end-labelling (TUNEL) assay was used to identify apoptotic cells with nuclear DNA fragmentation in brain perihematomal region. Staining was using a commercial one-step TUNEL apoptosis assay kit (Beyotime, C1090, Nanjing, China) and performed according to the manufacturer’s instructions. Following tissue immunofluorescence, the TUNEL reaction was performed. Brain sections incubation with TUNEL reaction mixture (1 h at 37 °C), then sections were washed with PBS and counterstained with DAPI. The colocalization of NeuN-TUNEL staining was examined using a Zeiss LSM 880 confocal microscope. TUNEL-positive neurons (NeuN positive) in the perihematomal region inside the striatum were quantified using ImageJ software on three different pictures per mouse taken under a 20X objective. The colocalization of GFAP-TUNEL staining was similar to that of NeuN-TUNEL staining.

### 4.19. In Vivo Blood-Brain Barrier Permeability Assay

In Vivo Blood-brain Barrier Permeability Assays were performed as described previously [63,64,65]. Briefly, control mice and ICH mice (±i.p. peptide) were intraperitoneally injected with FITC-labelled 4 kDa dextran (100 µL of 2 mM stock in PBS, Sigma Aldrich, 46944-100 MG-F, St Louis, MO, USA) after modelling 5 days, followed by anesthesia 5 min later. After a circulation time of 10 min for the tracer, the animals were prepared for transcardial perfusion, and ~ 300 µl blood was collected from the chest cavity just after atrial puncture, followed by perfusion for 3 min with PBS. Hemibrain (free of the olfactory lobes, cerebellum and hindbrain) and a single kidney were collected and immediately frozen in liquid nitrogen and stored at −80 °C. For fluorescence measurement, samples were thawed on ice, weighed and homogenized in PBS (400 µL for hemicerebrum and 600 µL for kidney), followed by centrifugation for 20 min at 4 °C and 15,000× *g*. Supernatants (100 µL) as well as equal volumes of serum (1/5 dilution in PBS) were loaded into a 96-well black plate, and fluorescence was measured at the corresponding excitation (490 nm)/emission (520 nm) in a plate reader (Tecan, Männedorf, Switzerland). Sham animals (without tracer injection) were used to subtract autofluorescence values. The permeability index (mL/g) was calculated as the ratio of tissue RFUs/g tissue weight to serum RFUs/mL serum.

### 4.20. Fluorescent Lectin Staining

To visualize brain microvessels, we used fluorescein-conjugated Lycopersicon esculentum lectin (1:200, Vector Laboratories, FL-1171, Burlingame, CA, USA) to immerse floating sections for 1 h. Sections were imaged on a Zeiss LSM 880 confocal microscope under a 20× objective, and images were processed with a 1.3 gamma setting. Three images were taken from 3 prespecified areas in the perihematomal region of the coronal brain per animal. Vessel branch points were quantified in each brain by a blinded investigator. A branch point was defined as a single fluorescein-labelled vessel noticeably separating into 2 distinct and separate vessel tracks [66].

### 4.21. Statistical Analysis

All experimental procedures were conducted blinded and performed 3 times repat independently as previously described [67]. Data are presented as the mean ± SEM. Statistical analysis was performed by One-way ANOVA, Two-way ANOVA and Student’s *t* test, as appropriate, using GraphPad Prism v7 (GraphPad Software Inc., San Diego, CA, USA) and SPSS software version 20.0 (SPSS, Inc., Chicago, IL, USA). The statistical parameters can be found in the relevant figure legends. Statistical significance was set at * *p* < 0.05; ** *p* < 0.01; *** *p* < 0.001; **** *p* < 0.0001.

## 5. Conclusions

The EGF3 domain of Netrin-1 is crucial to the interaction of Netrin-1 with DCC and can activate different signaling pathways. Truncated peptide E1 of Netrin-1 (residues 407–422) interacts with DCC to protect neurons from hemin-induced toxicity. The application of peptide E1 improves functional recovery in an experimental model of ICH. Furthermore, peptide E1 is able to protect neurons from degeneration and apoptosis after ICH.

## Supplementary Materials

The following are available online at https://www.mdpi.com/article/10.3390/ijms22094829/s1.

## References

[1] Kirshner H.S. (2008). Medical management of intracerebral haemorrhage: Back to the basics. Int. J. Clin. Pract..

[2] Anderson C.S., Heeley E., Huang Y., Wang J., Stapf C., Delcourt C., Lindley R., Robinson T., Lavados P., Neal B. (2013). Rapid blood-pressure lowering in patients with acute intracerebral hemorrhage. N. Engl. J. Med..

[3] Qureshi A.I., Mendelow A.D., Hanley D.F. (2009). Intracerebral haemorrhage. Lancet (Lond. Engl.).

[4] Keep R.F., Hua Y., Xi G. (2012). Intracerebral haemorrhage: Mechanisms of injury and therapeutic targets. Lancet Neurol..

[5] Schrag M., Kirshner H. (2020). Management of Intracerebral Hemorrhage: JACC Focus Seminar. J. Am. Coll. Cardiol..

[6] Kandasamy R., Idris Z., Abdullah J.M., July J., Wahjoepramono E.J. (2019). Surgery of Intracerebral Hemorrhage. Neurovascular Surgery Surgical Approaches for Neurovascular Diseases.

[7] Broughton B.R., Reutens D.C., Sobey C.G. (2009). Apoptotic mechanisms after cerebral ischemia. Stroke.

[8] Kristiansen M., Graversen J.H., Jacobsen C., Sonne O., Hoffman H.J., Law S.K., Moestrup S.K. (2001). Identification of the haemoglobin scavenger receptor. Nature.

[9] Madangarli N., Bonsack F., Dasari R., Sukumari-Ramesh S. (2019). Intracerebral Hemorrhage: Blood Components and Neurotoxicity. Brain Sci..

[10] Deiner M.S., Kennedy T.E., Fazeli A., Serafini T., Tessier-Lavigne M., Sretavan D.W. (1997). Netrin-1 and DCC mediate axon guidance locally at the optic disc: Loss of function leads to optic nerve hypoplasia. Neuron.

[11] Kennedy T.E., Serafini T., de la Torre J.R., Tessier-Lavigne M. (1994). Netrins are diffusible chemotropic factors for commissural axons in the embryonic spinal cord. Cell.

[12] Flores C. (2011). Role of netrin-1 in the organization and function of the mesocorticolimbic dopamine system. J. Psychiatry Neurosci. Jpn..

[13] Ren X.R., Ming G.L., Xie Y., Hong Y., Sun D.M., Zhao Z.Q., Feng Z., Wang Q., Shim S., Chen Z.F. (2004). Focal adhesion kinase in netrin-1 signaling. Nat. Neurosci..

[14] Zhang J.-H., Zhao Y.-F., He X.-X., Zhao Y., He Z.-X., Zhang L., Huang Y., Wang Y.-B., Hu L., Liu L. (2018). DCC-mediated Dab1 phosphorylation participates in the multipolar-to-bipolar transition of migrating neurons. Cell Rep..

[15] Ren X.R., Hong Y., Feng Z., Yang H.M., Mei L., Xiong W.C. (2008). Tyrosine phosphorylation of netrin receptors in netrin-1 signaling. Neurosignals.

[16] Cirulli V., Yebra M. (2007). Netrins: Beyond the brain. Nat. Rev. Mol. Cell Biol..

[17] Li W., Lee J., Vikis H.G., Lee S.H., Liu G., Aurandt J., Shen T.L., Fearon E.R., Guan J.L., Han M. (2004). Activation of FAK and Src are receptor-proximal events required for netrin signaling. Nat. Neurosci..

[18] Liu G., Beggs H., Jürgensen C., Park H.-T., Tang H., Gorski J., Jones K.R., Reichardt L.F., Wu J., Rao Y. (2004). Netrin requires focal adhesion kinase and Src family kinases for axon outgrowth and attraction. Nat. Neurosci..

[19] Finci L.I., Krüger N., Sun X., Zhang J., Chegkazi M., Wu Y., Schenk G., Mertens H.D.T., Svergun D.I., Zhang Y. (2014). The crystal structure of netrin-1 in complex with DCC reveals the bifunctionality of netrin-1 as a guidance cue. Neuron.

[20] Xu K., Wu Z., Renier N., Antipenko A., Tzvetkova-Robev D., Xu Y., Minchenko M., Nardi-Dei V., Rajashankar K.R., Himanen J. (2014). Neural migration. Structures of netrin-1 bound to two receptors provide insight into its axon guidance mechanism. Science.

[21] Lu H., Wang Y., He X., Yuan F., Lin X., Xie B., Tang G., Huang J., Tang Y., Jin K. (2012). Netrin-1 hyperexpression in mouse brain promotes angiogenesis and long-term neurological recovery after transient focal ischemia. Stroke.

[22] Guo D., Zhu Z., Zhong C., Peng H., Wang A., Xu T., Peng Y., Xu T., Chen C.S., Li Q. (2019). Increased Serum Netrin-1 Is Associated with Improved Prognosis of Ischemic Stroke. Stroke.

[23] Wu T.W., Li W.W., Li H. (2008). Netrin-1 attenuates ischemic stroke-induced apoptosis. Neuroscience.

[24] Navankasattusas S., Whitehead K.J., Suli A., Sorensen L.K., Lim A.H., Zhao J., Park K.W., Wythe J.D., Thomas K.R., Chien C.B. (2008). The netrin receptor UNC5B promotes angiogenesis in specific vascular beds. Development (Camb. Engl.).

[25] Bai L., Mei X., Wang Y., Yuan Y., Bi Y., Li G., Wang H., Yan P., Lv G. (2017). The Role of Netrin-1 in Improving Functional Recovery through Autophagy Stimulation Following Spinal Cord Injury in Rats. Front. Cell. Neurosci..

[26] Xie Z., Huang L., Enkhjargal B., Reis C., Wan W., Tang J., Cheng Y., Zhang J.H. (2017). Intranasal administration of recombinant Netrin-1 attenuates neuronal apoptosis by activating DCC/APPL-1/AKT signaling pathway after subarachnoid hemorrhage in rats. Neuropharmacology.

[27] Cui M.Z. (2015). Potential therapeutics for myocardial ischemia-reperfusion injury. Focus on “Induction of cardioprotection by small netrin-1-derived peptides”. Am. J. Physiol. Cell Physiol..

[28] Li Q., Cai H. (2015). Induction of cardioprotection by small netrin-1-derived peptides. Am. J. Physiol. Cell Physiol..

[29] Mazelin L., Bernet A., Bonod-Bidaud C., Pays L., Arnaud S., Gespach C., Bredesen D.E., Scoazec J.Y., Mehlen P. (2004). Netrin-1 controls colorectal tumorigenesis by regulating apoptosis. Nature.

[30] Mehlen P., Mazelin L. (2003). The dependence receptors DCC and UNC5H as a link between neuronal guidance and survival. Biol. Cell.

[31] Arakawa H. (2004). Netrin-1 and its receptors in tumorigenesis. Nat. Rev. Cancer.

[32] Chen J.-Y., He X.-X., Ma C., Wu X.-M., Wan X.-L., Xing Z.-K., Pei Q.-Q., Dong X.-P., Liu D.-X., Xiong W.-C. (2017). Netrin-1 promotes glioma growth by activating NF-κB via UNC5A. Sci. Rep..

[33] Yurchenco P.D., Wadsworth W.G. (2004). Assembly and tissue functions of early embryonic laminins and netrins. Curr. Opin. Cell Biol..

[34] Dun X.P., Parkinson D.B. (2017). Role of Netrin-1 Signaling in Nerve Regeneration. Int. J. Mol. Sci..

[35] Lai Wing Sun K., Correia J.P., Kennedy T.E. (2011). Netrins: Versatile extracellular cues with diverse functions. Development (Camb. Engl.).

[36] Tang T., Gao D., Yang X., Hua X., Li S., Sun H. (2019). Exogenous Netrin-1 Inhibits Autophagy of Ischemic Brain Tissues and Hypoxic Neurons via PI3K/mTOR Pathway in Ischemic Stroke. J. Stroke Cerebrovasc. Dis. Off. J. Natl. Stroke Assoc..

[37] Boyer N.P., Gupton S.L. (2018). Revisiting Netrin-1: One Who Guides (Axons). Front. Cell. Neurosci..

[38] Forcet C., Stein E., Pays L., Corset V., Llambi F., Tessier-Lavigne M., Mehlen P. (2002). Netrin-1-mediated axon outgrowth requires deleted in colorectal cancer-dependent MAPK activation. Nature.

[39] Guo Y.J., Pan W.W., Liu S.B., Shen Z.F., Xu Y., Hu L.L. (2020). ERK/MAPK signalling pathway and tumorigenesis. Exp. Ther. Med..

[40] Kefeli U., Ucuncu Kefeli A., Cabuk D., Isik U., Sonkaya A., Acikgoz O., Ozden E., Uygun K. (2017). Netrin-1 in cancer: Potential biomarker and therapeutic target?. Tumour Biol. J. Int. Soc. Oncodev. Biol. Med..

[41] Dang T.N., Robinson S.R., Dringen R., Bishop G.M. (2011). Uptake, metabolism and toxicity of hemin in cultured neurons. Neurochem. Int..

[42] Higdon A.N., Benavides G.A., Chacko B.K., Ouyang X., Johnson M.S., Landar A., Zhang J., Darley-Usmar V.M. (2012). Hemin causes mitochondrial dysfunction in endothelial cells through promoting lipid peroxidation: The protective role of autophagy. Am. J. Physiol. Heart Circ. Physiol..

[43] Wang J.J., Fu X.Q., Guo Y.G., Yuan L., Gao Q.Q., Yu H.L., Shi H.L., Wang X.Z., Xiong W.C., Zhu X.J. (2009). Involvement of headless myosin X in the motility of immortalized gonadotropin-releasing hormone neuronal cells. Cell Biol. Int..

[44] Guo Y., He X., Zhao L., Liu L., Song H., Wang X., Xu J., Ju X., Guo W., Zhu X. (2017). Gβ2 Regulates the Multipolar-Bipolar Transition of Newborn Neurons in the Developing Neocortex. Cereb. Cortex.

[45] Zhu X.J., Wang C.Z., Dai P.G., Xie Y., Song N.N., Liu Y., Du Q.S., Mei L., Ding Y.Q., Xiong W.C. (2007). Myosin X regulates netrin receptors and functions in axonal path-finding. Nat. Cell Biol..

[46] Wang J., Zhuang H., Doré S. (2006). Heme oxygenase 2 is neuroprotective against intracerebral hemorrhage. Neurobiol. Dis..

[47] Cai Y., Cho G.S., Ju C., Wang S.L., Ryu J.H., Shin C.Y., Kim H.S., Nam K.W., Jalin A.M., Sun W. (2011). Activated microglia are less vulnerable to hemin toxicity due to nitric oxide-dependent inhibition of JNK and p38 MAPK activation. J. Immunol..

[48] Min H., Choi B., Jang Y.H., Cho I.H., Lee S.J. (2017). Heme molecule functions as an endogenous agonist of astrocyte TLR2 to contribute to secondary brain damage after intracerebral hemorrhage. Mol. Brain.

[49] Wang J., Zhai W., Yu Z., Sun L., Li H., Shen H., Li X., Liu C., Chen G. (2017). Neuroprotection Exerted by Netrin-1 and Kinesin Motor KIF1A in Secondary Brain Injury following Experimental Intracerebral Hemorrhage in Rats. Front. Cell. Neurosci..

[50] Freitas C., Larrivee B., Eichmann A. (2008). Netrins and UNC5 receptors in angiogenesis. Angiogenesis.

[51] Castets M., Coissieux M.M., Delloye-Bourgeois C., Bernard L., Delcros J.G., Bernet A., Laudet V., Mehlen P. (2009). Inhibition of endothelial cell apoptosis by netrin-1 during angiogenesis. Dev. Cell.

[52] Lu X., Le Noble F., Yuan L., Jiang Q., De Lafarge B., Sugiyama D., Bréant C., Claes F., De Smet F., Thomas J.L. (2004). The netrin receptor UNC5B mediates guidance events controlling morphogenesis of the vascular system. Nature.

[53] Fazeli A., Dickinson S.L., Hermiston M.L., Tighe R.V., Steen R.G., Small C.G., Stoeckli E.T., Keino-Masu K., Masu M., Rayburn H. (1997). Phenotype of mice lacking functional Deleted in colorectal cancer (Dcc) gene. Nature.

[54] He Z.X., Song H.F., Liu T.Y., Ma J., Xing Z.K., Yin Y.Y., Liu L., Zhang Y.N., Zhao Y.F., Yu H.L. (2019). HuR in the Medial Prefrontal Cortex is Critical for Stress-Induced Synaptic Dysfunction and Depressive-Like Symptoms in Mice. Cereb. Cortex.

[55] Kruger R.P., Lee J., Li W., Guan K.L. (2004). Mapping netrin receptor binding reveals domains of Unc5 regulating its tyrosine phosphorylation. J. Neurosci..

[56] Ma J., Zhang L.Q., He Z.X., He X.X., Wang Y.J., Jian Y.L., Wang X., Zhang B.B., Su C., Lu J. (2019). Autism candidate gene DIP2A regulates spine morphogenesis via acetylation of cortactin. PLoS Biol..

[57] Alim I., Caulfield J.T., Chen Y., Swarup V., Geschwind D.H., Ivanova E., Seravalli J., Ai Y., Sansing L.H., Ste Marie E.J. (2019). Selenium Drives a Transcriptional Adaptive Program to Block Ferroptosis and Treat Stroke. Cell.

[58] Li Z., Chen-Roetling J., Regan R.F. (2009). Increasing expression of H- or L-ferritin protects cortical astrocytes from hemin toxicity. Free Radic. Res..

[59] Kang M.K., Kim T.J., Kim Y.J., Kang L., Kim J., Lee N., Hyeon T., Lim M.S., Mo H.J., Shin J.H. (2020). Targeted Delivery of Iron Oxide Nanoparticle-Loaded Human Embryonic Stem Cell-Derived Spherical Neural Masses for Treating Intracerebral Hemorrhage. Int. J. Mol. Sci..

[60] Krafft P.R., Rolland W.B., Duris K., Lekic T., Campbell A., Tang J., Zhang J.H. (2012). Modeling intracerebral hemorrhage in mice: Injection of autologous blood or bacterial collagenase. J. Vis. Exp. Jove.

[61] Karuppagounder S.S., Alim I., Khim S.J., Bourassa M.W., Sleiman S.F., John R., Thinnes C.C., Yeh T.L., Demetriades M., Neitemeier S. (2016). Therapeutic targeting of oxygen-sensing prolyl hydroxylases abrogates ATF4-dependent neuronal death and improves outcomes after brain hemorrhage in several rodent models. Sci. Transl. Med..

[62] Mao Y., Black A.M.B., Milbourn H.R., Krakonja S., Nesbit M., Bartlett C.A., Fehily B., Takechi R., Yates N.J., Fitzgerald M. (2018). The Effects of a Combination of Ion Channel Inhibitors in Female Rats Following Repeated Mild Traumatic Brain Injury. Int. J. Mol. Sci..

[63] Devraj K., Guérit S., Macas J., Reiss Y. (2018). An In Vivo Blood-brain Barrier Permeability Assay in Mice Using Fluorescently Labeled Tracers. J. Vis. Exp. Jove.

[64] Devraj G., Guérit S., Seele J., Spitzer D., Macas J., Khel M.I., Heidemann R., Braczynski A.K., Ballhorn W., Günther S. (2020). HIF-1α is involved in blood-brain barrier dysfunction and paracellular migration of bacteria in pneumococcal meningitis. Acta Neuropathol..

[65] Vázquez-Rosa E., Shin M.K., Dhar M., Chaubey K., Cintrón-Pérez C.J., Tang X., Liao X., Miller E., Koh Y., Barker S. (2020). P7C3-A20 treatment one year after TBI in mice repairs the blood-brain barrier, arrests chronic neurodegeneration, and restores cognition. Proc. Natl. Acad. Sci. USA.

[66] Venna V.R., Li J., Hammond M.D., Mancini N.S., McCullough L.D. (2014). Chronic metformin treatment improves post-stroke angiogenesis and recovery after experimental stroke. Eur. J. Neurosci..

[67] He Z.X., Yin Y.Y., Xi K., Xing Z.K., Cao J.B., Liu T.Y., Liu L., He X.X., Yu H.L., Zhu X.J. (2020). Nucleus Accumbens Tac1-Expressing Neurons Mediate Stress-Induced Anhedonia-like Behavior in Mice. Cell Rep..

