# Supplementary material for: A Novel Netrin-1-Derived Peptide Enhances Protection against Neuronal Death and Mitigates of Intracerebral Hemorrhage in Mice
Source: Int J Mol Sci. 2021 May 2;22(9):4829. doi: 10.3390/ijms22094829 (PMC8125294; doi:10.3390/ijms22094829)
Supplement: Supplementary file 1 [file ijms-22-04829-s001.zip › ijms-1204681-supplementary.pdf]

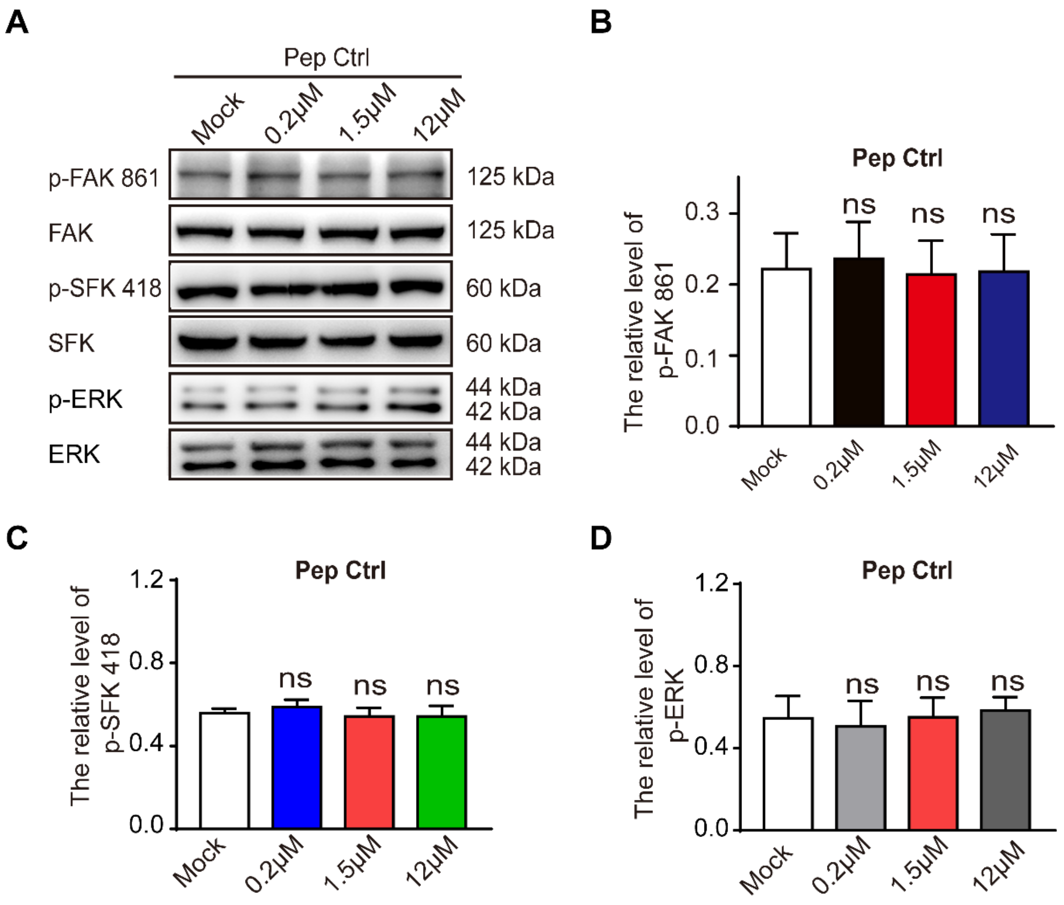

Figure S1. Pep Ctrl does not activate downstream pathways of Netrin-1. (A) Cortical neurons were stimulated with gradient concentration of Pep Ctrl. Cell lysates were incubated with the indicated antibodies to detect the phosphorylation of FAK, SFK and ERK. (B) Quantifications of peptide induced FAK PY861 in neurons. n = 3. (C) Quantifications of peptide induced SFK PY418 in neurons. n = 3. (C) Quantifications of peptide induced ERK PY202/204 in neurons. n = 3. Date are presented as the means ± SEM. One-way ANOVA was used for all statistical analyses shown in this figure (ns, not significant).

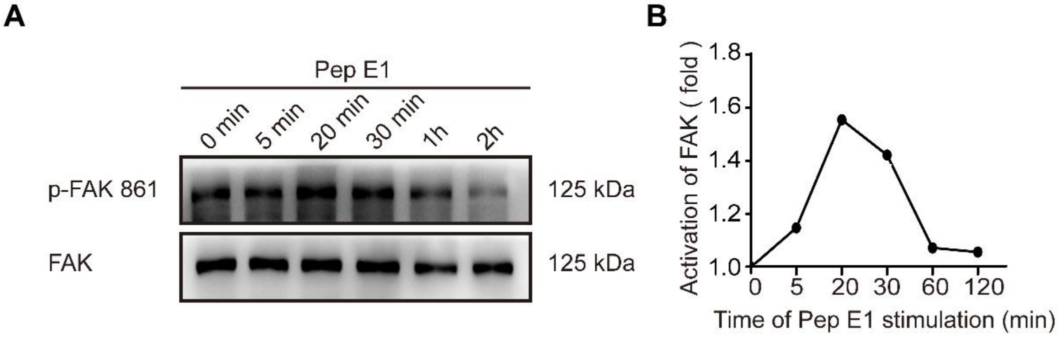

Figure S2. Pep E1 increased FAK tyrosine phosphorylation in a time-dependent manner.
(A) Cortical neurons (DIV3) were stimulated with peptide E1 for the indicated time. Cell lysates were incubated with the specially antibody to detect the phosphorylation of FAK Y861. (B) Quantifications of peptide E1 induced FAK PY861 in neurons were shown in (A). n = 3.

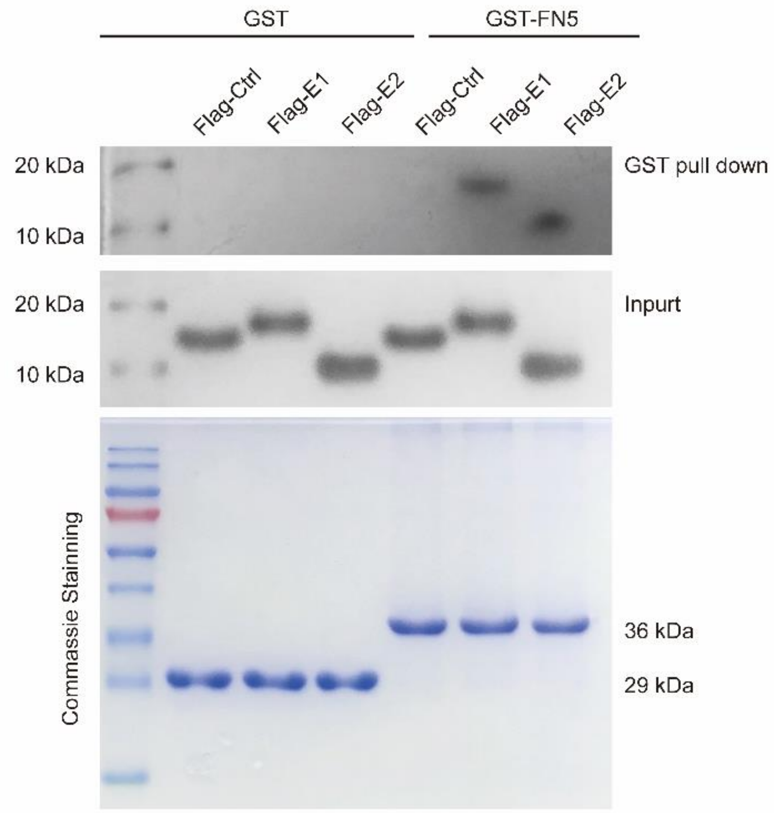

Figure S3. Pep E1 and E2 direct binding with DCC's FN5 domain. Synthesized N-terminal added Flag sequence peptides Flag-Ctrl, Flag-E1 and Flag-E2 were incubated with the GST-DCC-FN5 and GST proteins respectly, then evaluated by SDS-PAGE.

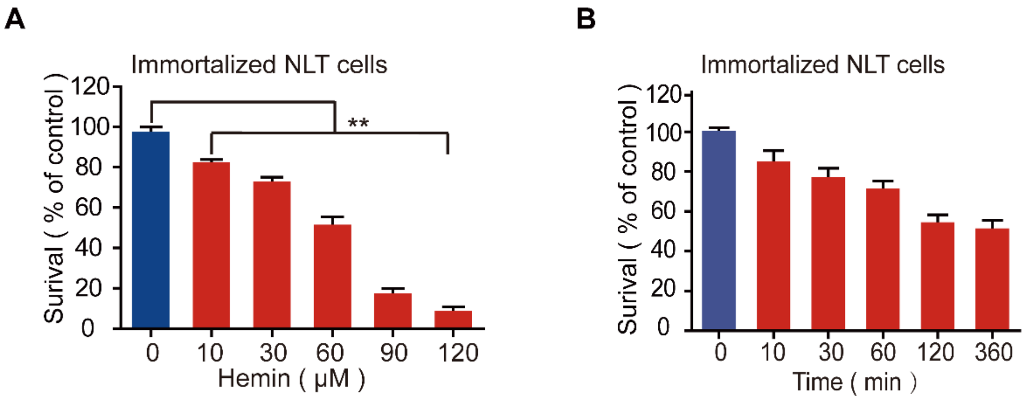

Figure S4. Hemin induced a dose and time-dependent neurotoxicity in cultured NLT cells. (A) CCK8 assays showed that hemin induces dose-dependent death of NLT cells. n = 4. (B) CCK8 assays showed that hemin induces time-dependent death of NLT cells. n = 4. Date are presented as the means ± SEM. One-way ANOVA was used for all statistical analyses shown in this figure (***p* < 0.001, ns, not significant).

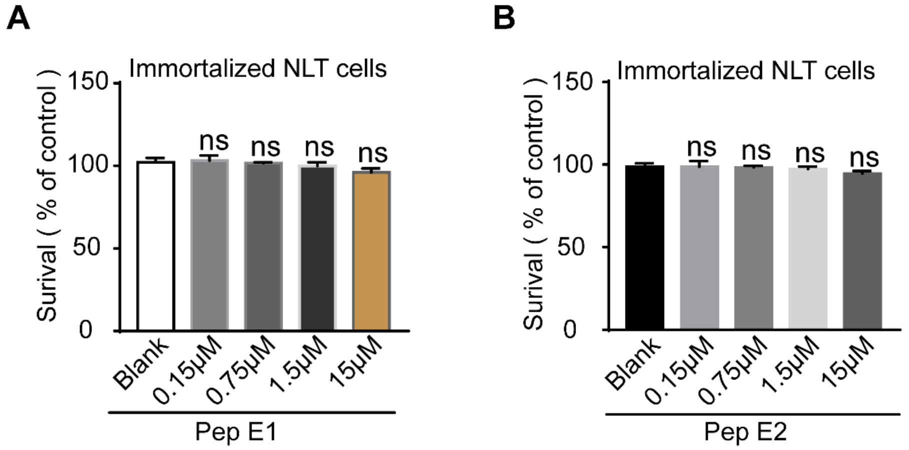

Figure S5. The peptides don't affect cell viability in NLT cells. (A) CCK8 assays showed that series dose of Pep E1 has not impact on cell viability. n = 4. (B) CCK8 assays showed that series dose of Pep E2 does not affect cell viability. n = 4. Date are presented as the means ± SEM. One-way ANOVA was used for all statistical analyses shown in this figure (ns, not significant).

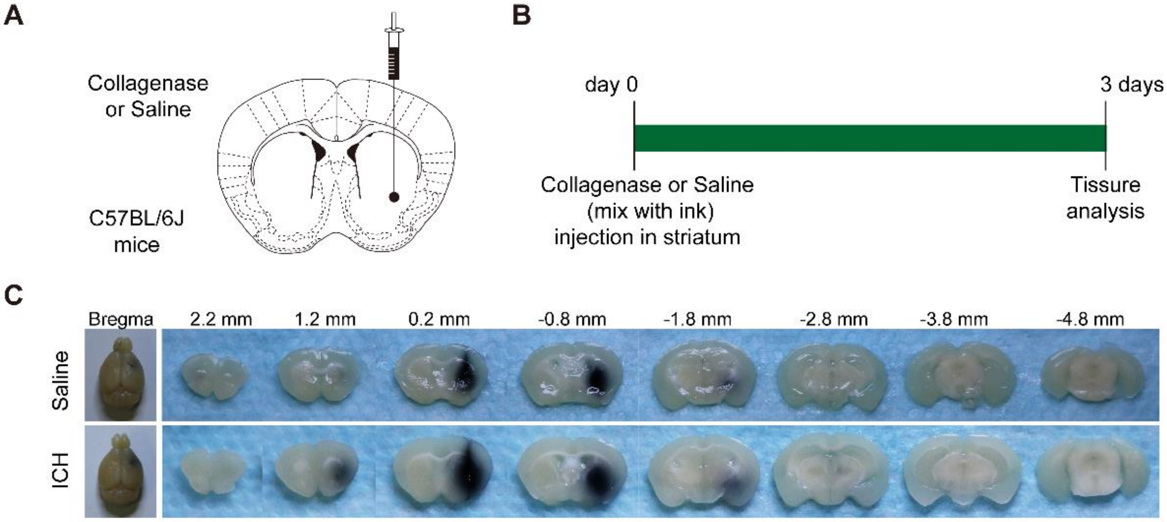

Figure S6. Collagenase injection volume is measured in mouse model of ICH. (A) Schematic of strategies used to inject Collagenase and Saline in C57 mice. (B) Experimental timeline. (C) Representative images showing ink area were similar in the Saline and ICH brain.

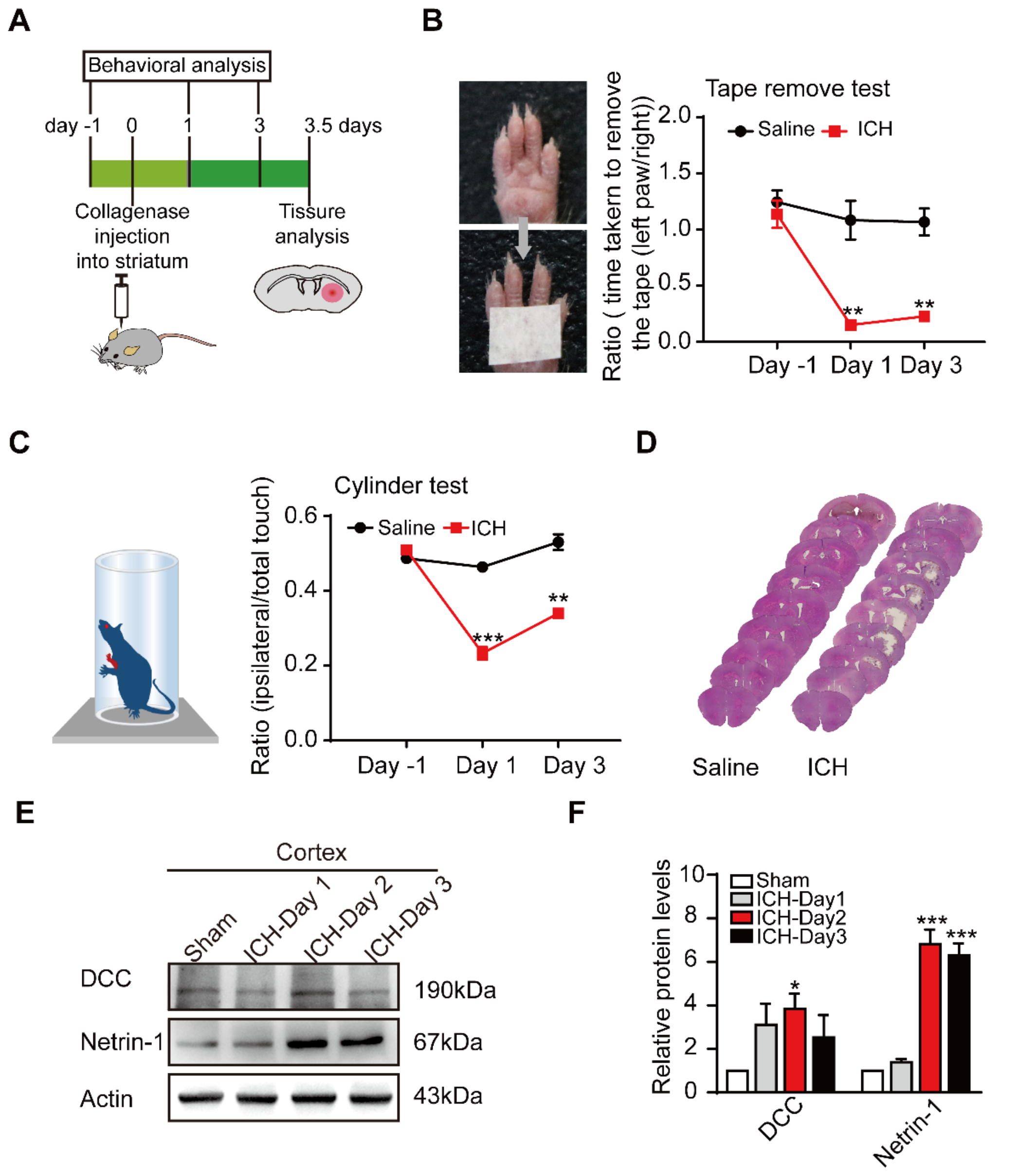

Figure S7. The protein levels of Netrin-1 and DCC is increased following experimental ICH. (A) Schematic of the experimental paradigm of modeling ICH in mice. (B-C) Intracerebral hemorrhage in mice were shown neurological deficit by an adhesive tape remove test (B) and cylinder test (C). n = 3. (D) Representative HE stained brain sections at 3 days after ICH built. Scale bar, 1 mm. (E) Immunoblot analysis of Netrin-1and DCC in the ipsilateral cortex at different time points after ICH. (F) Quantitative analysis of the western blot results shown in (E), respectively (**p* < 0.05, ***p* < 0.01, ****p* < 0.001; ns, not significant). n = 3.

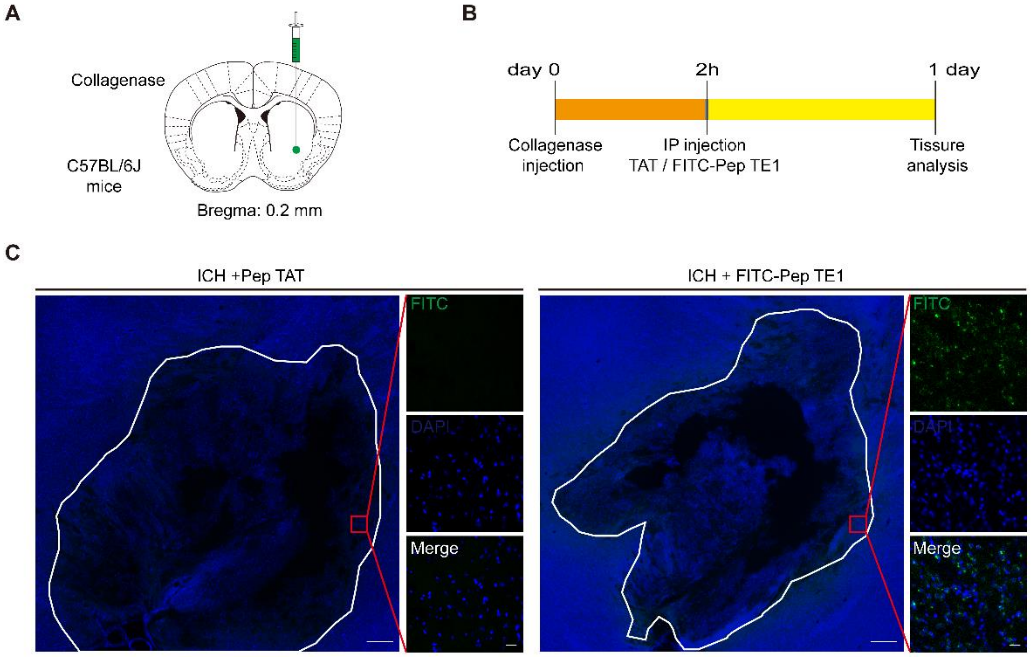

Figure S8. The FITC signal of FITC labeled peptide TE1 was detectable in the hematoma region after ICH. (A) Schematic of strategies used to inject Collagenase and Saline in C57 mice. (B) Experimental timeline. (C) Representative images detective FITC signals of hematoma region. Scale bars, 200 and 10 μm respectively.

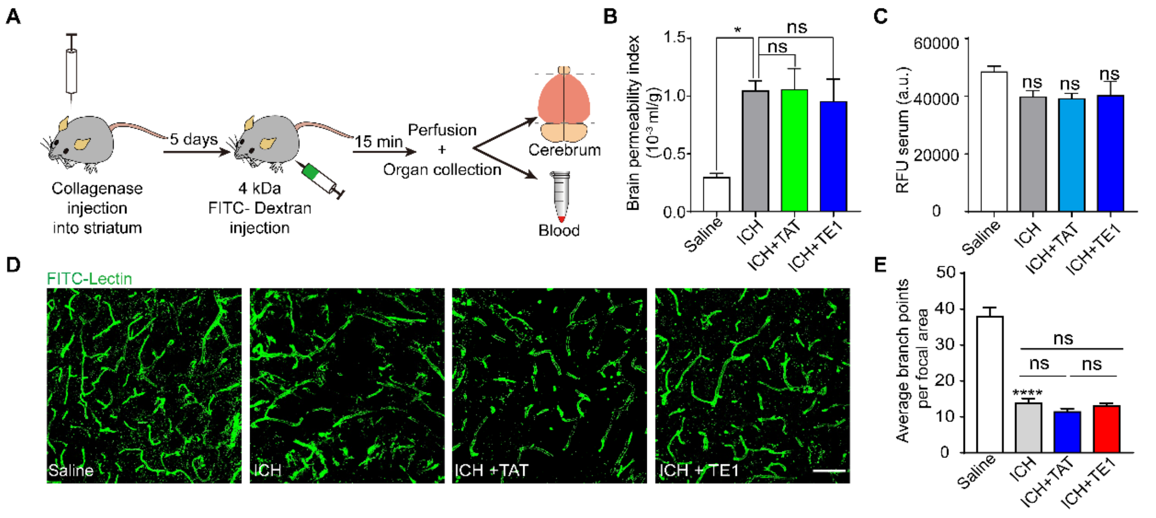

Figure S9. Netrin-1 derived peptide E1 no effected on the disruption of the blood-brain barrier permeability and microvessel after ICH. (A) Illustrations of in vivo BBB permeability assay using fluorescent tracers in an intracerebral hemorrhage model. (B) Homogenized ipsilateral hemisphere (free of the olfactory lobes, cerebellum and hindbrain) were utilized to obtain dextran permeability by measuring the raw fluorescence units (RFUs) on a microplate reader. Set the gain to optimal and use excitation/emission (nm) values of 490/520 for FITC dye and start the measurement to obtain the RFUs. Permeability Index (mL/g) = (Tissue RFUs/g tissue weight) / (Serum RFUs/mL serum). n = 3. (C) Serum fluorescence values (arbitrary units—a.u.) indicate equivalent tracer absorption between treat with/without TE1 mice. n = 3. (D) Representative confocal microscopy analysis of lectin-positive microvessels (green) in mice treat with/without TE1 at 5 days after ICH. Scale bar, 50 μm. (E) Quantitative analyses for the total number of branch points in the focal area between with/without TE1 treatment groups of the perihematomal region. n = 4.

Supplementary Table 1

Figure	Measure	Value	Comparisons	Analysis	Statistic value	P value
Figure. 2C	protein level	Mock = 0.5027<br>Netrin-1 = 0.9411<br>Netrin-1-(△407-443) = 0.627	Mock (n = 3) VS<br>Netrin-1 (n = 3)<br>Netrin-1-(△407-443) (n = 3)	One-way ANOVA	F (2, 6) = 9.728	Mock vs. NTN1-WT: p = 0.0123<br>Mock vs. NTN1-（△407-443): p = 0.0123<br>NTN1-WT vs. NTN1-（△407-443）: p= 0.0502
Figure. 2E	protein level	GST = 0.2912<br>GST-EGF3 = 0.6181<br>Netrin-1 = 0.8123	GST (n = 3) VS<br>GST-EGF3 (n = 3)<br>Netrin-1 (n = 3)	One-way ANOVA	F (2, 6) = 47.77	GST vs GST-EGF3: p = 0.022<br>GST vs Netrin-1: p = 0.002<br>GST-EGF3 vs Netrin-1: p = 0.0263
Figure. 2F	protein level	GST = 0.6445<br>GST-EGF3 = 0.8867<br>Netrin-1 = 1.019	GST (n = 3) VS<br>GST-EGF3 (n = 3)<br>Netrin-1 (n = 3)	One-way ANOVA	F (2, 6) = 16.42	GST vs GST-EGF3: p = 0.0249<br>GST vs Netrin-1: p = 0.0032<br>GST-EGF3 vs Netrin-1: p = 0.1930
Figure. 2G	protein level	GST = 0.3535<br>GST-EGF3 = 0.7297<br>Netrin-1 = 0.9449	GST (n = 3) VS<br>GST-EGF3 (n = 3)<br>Netrin-1 (n = 3)	One-way ANOVA	F (2, 6) = 16.25	GST vs GST-EGF3: p = 0.0269<br>GST vs Netrin-1: p = 0.0032<br>GST-EGF3 vs Netrin-1: p = 0.1813
Figure. 3D	protein level	Mock = 0.3546<br>0.2 µM = 0.3569<br>1.5 µM = 0.8771<br>12 µM = 0.4401	Mock (n = 3) VS<br>0.2 µM (n = 3)<br>1.5 µM (n = 3)<br>12 µM (n = 3)	One-way ANOVA	F (3, 8) = 4.704	Mock vs 0.2 µM: p = 0.9999<br>Mock vs 1.5 µM: p = 0.0309<br>Mock vs 12 µM: p = 0.9126
Figure. 3E	protein level	Mock = 0.3793<br>0.2 µM = 0.3584<br>1.5 µM = 0.8537<br>12 µM = 0.4884	Mock (n = 3) VS<br>0.2 µM (n = 3)<br>1.5 µM (n = 3)<br>12 µM (n = 3)	One-way ANOVA	F (3, 8) = 3.996	Mock vs 0.2 µM: p = 0.9983<br>Mock vs 1.5 µM: p = 0.0471<br>Mock vs 12 µM: p = 0.8420
Figure. 3F	protein level	Mock = 0.508<br>0.2 µM = 0.533<br>1.5 µM = 0.5329<br>12 µM = 0.5501	Mock (n = 3) VS<br>0.2 µM (n = 3)<br>1.5 µM (n = 3)<br>12 µM (n = 3)	One-way ANOVA	F (3, 8) = 0.0379	Mock vs 0.2 µM: p = 0.9941<br>Mock vs 1.5 µM: p = 0.9942<br>Mock vs 12 µM: p = 0.9739
Figure. 3G	protein level	Mock = 0.4042<br>0.2 µM = 0.569<br>1.5 µM = 0.9628<br>12 µM = 0.9009	Mock (n = 3) VS<br>0.2 µM (n = 3)<br>1.5 µM (n = 3)<br>12 µM (n = 3)	One-way ANOVA	F (3, 8) = 5.096	Mock vs 0.2 µM: p = 0.6529<br>Mock vs 1.5 µM: p = 0.0255<br>Mock vs 12 µM: p = 0.0437
Figure. 3H	protein level	Mock = 0.426<br>0.2 µM = 0.518	Mock (n = 3) VS<br>0.2 µM (n = 3)	One-way ANOVA	F (3, 8) = 8.01	Mock vs 0.2 µM: p = 0.7523<br>Mock vs 1.5 µM: p = 0.0104

		1.5 μM = 0.8684<br>12 μM = 0.8353	1.5 μM (n = 3)<br>12 μM (n = 3)			Mock vs 12 μM: p = 0.0158
Figure. 3I	protein level	Mock = 0.3051<br>0.2 μM = 0.6627<br>1.5 μM = 1.105<br>12 μM = 0.9556	Mock (n = 3) VS<br>0.2 μM (n = 3)<br>1.5 μM (n = 3)<br>12 μM (n = 3)	One-way ANOVA	F (3, 8) = 4.889	Mock vs 0.2 μM: p = 0.3252<br>Mock vs 1.5 μM: p = 0.0190<br>Mock vs 12 μM: p = 0.0499
Figure. 3K $DCC^{+/+}$	protein level	Pep Ctrl = 0.2937<br>Netrin-1 = 0.9328<br>Pep E1 = 0.8503<br>Pep E2 = 0.8005	Pep Ctrl (n = 3) VS<br>Netrin-1 (n = 3)<br>Pep E1 (n = 3)<br>Pep E2 (n = 3)	One-way ANOVA	F(3, 8) = 5.586	Pep ctrl vs Netrin-1: p = 0.0153<br>Pep ctrl vs Pep E1: p = 0.0303<br>Pep ctrl vs Pep E1: p = 0.0463
Figure. 3K $DCC^{-/-}$	protein level	Pep Ctrl = 0.2927<br>Netrin-1 = 0.5313<br>Pep E1 = 0.4887<br>Pep E2 = 0.4615	Pep Ctrl (n = 3) VS<br>Netrin-1 (n = 3)<br>Pep E1 (n = 3)<br>Pep E2 (n = 3)	One-way ANOVA	F(3, 8) = 0.3429	Pep ctrl vs Netrin-1: p = 0.6786<br>Pep ctrl vs Pep E1: p = 0.7825<br>Pep ctrl vs Pep E1: p = 0.8434
Figure. 4B DCC	protein level	Sham = 1<br>Hemin-2h = 1.963<br>Hemin-6h = 6.563<br>Hemin-12h = 5.703<br>Hemin-24h = 1.891	Sham (n = 3) VS<br>Hemin-2h (n = 3)<br>Hemin-6h (n = 3)<br>Hemin-12h (n = 3)<br>Hemin-24h (n = 3)	One-way ANOVA	F(4, 10) = 12.79	Sham vs Hemin-2h: p = 0.7397<br>Sham vs Hemin-6h: p = 0.0008<br>Sham vs Hemin-12h: p = 0.0028<br>Sham vs Hemin-24 h: p = 0.7842
Figure. 4B Netrin-1	protein level	Sham = 1<br>Hemin-2h = 1.976<br>Hemin-6h = 5.731<br>Hemin-12h = 4.426<br>Hemin-24h = 3.597	Sham (n = 3) VS<br>Hemin-2h (n = 3)<br>Hemin-6h (n = 3)<br>Hemin-12h (n = 3)<br>Hemin-24h (n = 3)	One-way ANOVA	F(4, 10) = 2.873	Sham vs Hemin-2h: p = 0.9230<br>Sham vs Hemin-6h: p = 0.0419<br>Sham vs Hemin-12h: p = 0.1581<br>Sham vs Hemin-24 h: p = 0.3418
Figure. 4C	Surival (% of control)	Blank = 97.49<br>Pep Ctrl = 97.64<br>Pep E1 = 99.92<br>Pep E2 = 97.54	Blank (n = 4) VS<br>Pep Ctrl (n = 4)<br>Pep E1 (n = 4)<br>Pep E2 (n = 4)	One-way ANOVA	F(3, 12) = 0.2046	Blank vs Pep Ctrl: p > 0.9999<br>Blank vs Pep E1: p = 0.9110<br>Blank vs Pep E2: p > 0.9999
Figure. 4D	Surival (% of control)		Hemin+Pep Ctrl (n =4) VS<br>Hemin+Netrin-1 (n =4)<br>Hemin+Pep E1 (n =4)<br>Hemin+Pep E2 (n =4)	Two-way ANOVA	F (9, 48) = 1.728<br>F (3, 48) = 2.143<br>F (3, 48) = 10.77	P=0.1083<br>P=0.1071<br>P<0.0001
Figure. 4F	% of Cell viability	Blank = 97.15	Blank (n = 9)			Hemin vs blank: p = 0.0001

		Hemin = 56.35<br>Hemin+Netrin-1 = 57.81<br>Hemin+ Ctrl = 57.81<br>Hemin+ E1 = 72.91<br>Hemin+E2 = 55.49	Hemin (n = 9) VS<br>Hemin+Netrin-1 (n = 9)<br>Hemin+Ctrl (n = 9)<br>Hemin+E1 (n = 9)<br>Hemin+E2 (n = 9)	One-way ANOVA	F(5, 48) = 24.98	Hemin vs Hemin+Netrin-1: p = 0.036<br>Hemin vs Hemin+Ctrl: p = 0.3227<br>Hemin vs Hemin+E1: p = 0.030<br>Hemin vs Hemin+E2: p = 0.9997
Figure. 4G	% of Dead cells	Blank = 2.855<br>Hemin = 43.85<br>Hemin+Netrin-1 = 27.37<br>Hemin+ Ctrl = 42.19<br>Hemin+ E1 = 27.09<br>Hemin+E2 = 43.92	Blank (n = 9)<br>Hemin (n = 9)<br>Hemin+Netrin-1 (n = 9)<br>Hemin+Ctrl (n = 9)<br>Hemin+E1 (n = 9)<br>Hemin+E2 (n = 9)	One-way ANOVA	F(5, 48) = 23.34	Hemin vs blank: p = 0.0001<br>Hemin vs Hemin+Netrin-1: p = 0.043<br>Hemin vs Hemin+Ctrl: p = 0.9961<br>Hemin vs Hemin+E1: p = 0.036<br>Hemin vs Hemin+E2: p = 0.9999
Figure. 4H	Surival (% of control)	Blank = 95<br>Hemin = 52.5<br>Hemin+0.15 µM = 63.01<br>Hemin+ 5 µM = 60.69<br>Hemin+ 10 µM = 56	Blank (n = 8)<br>Hemin (n = 8) VS<br>Hemin+0.15 µM (n = 8)<br>Hemin+ 5 µM (n = 8)<br>Hemin+ 10 µM (n = 8)	One-way ANOVA	F(4, 35) = 16.02	Hemin vs blank: p = 0.0001<br>Hemin vs Hemin+0.15 µM: p = 0.048<br>Hemin vs Hemin+ 5 µM: p = 0.4669<br>Hemin vs Hemin+ 10 µM: p = 0.9395
Figure. 5C	Weight chang (% of control)	Saline = 101.8<br>ICH = 92.19<br>ICH+TAT = 90.4<br>ICH+TE1 = 98.48	Saline (n = 6) VS<br>ICH (n = 6)<br>ICH+TAT (n = 6)<br>ICH+te1 (n = 6)	One-way ANOVA	F(3, 20) = 11.78	Sham vs ICH: p = 0.008<br>Sham vs ICH+TAT: p = 0.001<br>Sham vs. ICH+TE1: p = 0.3297
Figure. 5E	Remove tape ratio		Saline (n = 10)<br>ICH (n = 6) VS<br>ICH+TAT (n = 6)<br>ICH+te1 (n = 8)	Two-way ANOVA	F (9, 106) = 6.354<br>F (3, 106) = 38.08<br>F (3, 106) = 45.53	P<0.0001<br>P<0.0001<br>P<0.0001
Figure. 5F	Touch ratio		Saline (n = 10)<br>ICH (n = 6) VS	Two-way ANOVA	F (9, 92) = 44.09<br>F (3, 92) = 263.3	P<0.0001<br>P<0.0001

			ICH+TAT (n = 6)<br>ICH+te1 (n = 8)		F (3, 92) = 260.9	P<0.0001
Figure. 5H	Hemorrhage volume	Saline = 0.012<br>ICH = 7.605<br>ICH+TAT = 7.6375<br>ICH+TE1 = 4.3875	Saline (n = 10) VS<br>ICH (n = 6)<br>ICH+TAT (n = 6)<br>ICH+te1 (n = 8)	One-way ANOVA	F(3, 49) = 32.78	saline vs ICH: p < 0.0001<br>saline vs ICH+TAT p < 0.0001<br>saline vs ICH+TE1: p < 0.0001<br>ICH vs ICH+TAT: p > 0.9999<br>ICH vs ICH+TE1: p = 0.0031<br>ICH+TAT vs ICH+TE1: p = 0.0085
Figure. 6B	Cell number	Saline = 3.083<br>ICH = 78.38<br>ICH+TAT = 77.49<br>ICH+TE1 = 30.02	Saline (n =4) VS<br>ICH (n = 4)<br>ICH+TAT (n = 4)<br>ICH+te1 (n = 4)	One-way ANOVA	F(3, 158) = 54.53	saline vs ICH: p < 0.0001<br>saline vs ICH+TAT p < 0.0001<br>saline vs ICH+TE1: p = 0.0023<br>ICH vs ICH+TAT: p = 0.9989<br>ICH vs ICH+TE1: p = 0.0041<br>ICH+TAT vs ICH+TE1: p = 0.0032
Figure. 6D	Cell number	Saline = 11.53<br>ICH = 52.22<br>ICH+TAT = 54.22<br>ICH+TE1 = 42.86	Saline (n =4) VS<br>ICH (n = 4)<br>ICH+TAT (n = 4)<br>ICH+te1 (n = 4)	One-way ANOVA	F(3, 99) = 36.52	saline vs ICH: p < 0.0001<br>saline vs ICH+TAT p < 0.0001<br>saline vs ICH: p < 0.0001<br>ICH vs ICH+TAT: p = 0.9410<br>ICH vs ICH+TE1: p = 0.0328<br>ICH+TAT vs ICH+TE1: p = 0.0132
Figure. 6F	Cell number	Saline = 7.585<br>ICH = 9.731<br>ICH+TAT = 11.1<br>ICH+TE1 = 9.458	Saline (n =4) VS<br>ICH (n = 4)<br>ICH+TAT (n = 4)<br>ICH+te1 (n = 4)	One-way ANOVA	F(3, 84) = 1.2	saline vs ICH: p = 0.6329<br>saline vs ICH+TAT p = 0.2465<br>saline vs ICH: p = 0.8343<br>ICH vs ICH+TAT: p = 0.7667<br>ICH vs ICH+TE1: p = 0.9989<br>ICH+TAT vs ICH+TE1: p = 0.8288
Figure. S1B	protein level	Mock = 0.2218<br>0.2 μM = 0.2363<br>1.5 μM = 0.2143<br>12 μM = 0.218	Mock (n = 3) VS<br>0.2 μM (n = 3)<br>1.5 μM (n = 3)<br>12 μM (n = 3)	One-way ANOVA	F (3, 8) = 0.03619	Mock vs 0.2 μM: p = 0.9937<br>Mock vs 1.5 μM: p = 0.9991<br>Mock vs 12 μM: p = 0.9999
Figure. S1C	protein level	Mock = 0.5585<br>0.2 μM = 0.5885<br>1.5 μM = 0.5425	Mock (n = 3) VS<br>0.2 μM (n = 3)<br>1.5 μM (n = 3)	One-way ANOVA	F (3, 8) = 0.3093	Mock vs 0.2 μM: p = 0.9050<br>Mock vs 1.5 μM: p = 0.9827<br>Mock vs 12 μM: p = 0.9817

		12 μM = 0.5422	12 μM (n = 3)			
Figure. S1D	protein level	Mock = 0.5463<br>0.2 μM = 0.507<br>1.5 μM = 0.552<br>12 μM = 0.5833	Mock (n = 3) VS<br>0.2 μM (n = 3)<br>1.5 μM (n = 3)<br>12 μM (n = 3)	One-way ANOVA	F (3, 8) = 0.0974	Mock vs 0.2 μM: p = 0.9845<br>Mock vs 1.5 μM: p = 0.9999<br>Mock vs 12 μM: p = 0.9871
Figure. S4A	Surival (% of control)	0 μM = 98.2<br>10 μM = 83.01<br>30 μM = 73.67<br>60 μM = 52.23<br>90 μM = 18.32<br>120 μM = 9.669	0 μM (n = 4) VS<br>10 μM (n = 4)<br>30 μM (n = 4)<br>60 μM (n = 4)<br>90 μM (n = 4)<br>120 μM (n = 4)	One-way ANOVA	F (5, 18) = 369.1	0 μM vs 10 μM hemin: p < 0.0001<br>0 μM vs 30 μM hemin: p < 0.0001<br>0 μM vs 60 μM hemin: p < 0.0001<br>0 μM vs 90 μM hemin: p < 0.0001<br>0 μM vs 120 μM hemin: p < 0.0001<br>10 μM hemin vs 120 μM hemin: p = 0.0040
Figure. S4B	Surival (% of control)	0 min = 101.2<br>10 min = 85.78<br>30 min = 77.93<br>60 min = 72.35<br>120 min = 55.38<br>360 min = 52.23	0 min (n = 4) VS<br>10 min (n = 4)<br>30 min (n = 4)<br>60 min (n = 4)<br>120 min (n = 4)<br>360 min (n = 4)	One-way ANOVA	F (5, 18) = 31.71	blank vs 10min: p = 0.0159<br>blank vs 30min: p = 0.0004<br>blank vs 60min: p = 0.0001<br>blank vs 120min: p = 0.0001<br>blank vs 360min: p = 0.0001
Figure. S5A	Surival (% of control)	Blank = 102.2<br>0.15 μM = 103.2<br>0.75 μM = 101.6<br>1.5 μM = 99.92<br>15 μM = 96.1	Blank (n = 4) VS<br>0.15 μM (n = 4)<br>0.75 μM (n = 4)<br>1.5 μM (n = 4)<br>15 μM (n = 4)	One-way ANOVA	F (4, 15) = 1.438	Blank vs 0.15 μM: p = 0.9928<br>Blank vs 0.75 μM: p = 0.9992<br>Blank vs 1.5 μM: p = 0.8963<br>Blank vs 12 μM: p = 0.2382
Figure. S5B	Surival (% of control)	Blank = 98.63<br>0.15 μM = 98.7<br>0.75 μM = 98.27<br>1.5 μM =97.29<br>15 μM = 94.58	Blank (n = 4) VS<br>0.15 μM (n = 4)<br>0.75 μM (n = 4)<br>1.5 μM (n = 4)<br>15 μM (n = 4)	One-way ANOVA	F (4, 15) = 1.438	Blank vs 0.15 μM: p = 0.9999<br>Blank vs 0.75 μM: p = 0.9974<br>Blank vs 1.5 μM: p = 0.7885<br>Blank vs 12 μM: p = 0.0559
Figure. S7B	Remove tape ratio		Saline (n = 3) VS<br>ICH (n = 3)	Two-way ANOVA	F (2, 12) = 6.64<br>F (2, 12) = 11.97<br>F (1, 12) = 19.4	P=0.0114<br>P=0.0014<br>P=0.0009
Figure. S7C	Touch ratio		Saline (n = 3) VS	Two-way ANOVA	F (2, 12) = 57.08	P<0.0001

			ICH (n = 3)		F (2, 12) = 69.26<br>F (1, 12) = 162.8	P<0.0001<br>P<0.0001
Figure. S7E<br>DCC	protein level	Sham = 1<br>ICH-Day 1 = 3.449<br>ICH-Day 2 = 4.11<br>ICH-Day 3 = 2.529	Sham (n = 3) VS<br>ICH-Day 1 (n = 3)<br>ICH-Day 2 (n = 3)<br>ICH-Day 3 (n = 3)	One-way ANOVA	F(3, 8) = 3.539	Sham vs ICH-Day 1: p = 0.0993<br>Sham vs. ICH-Day 2: p = 0.0378<br>Sham vs. ICH-Day 3: p = 0.3587
Figure. S7F<br>Netrin-1	protein level	Sham = 1<br>ICH-Day 1 = 1.4<br>ICH-Day 2 = 5.821<br>ICH-Day 3 = 5.293	Sham (n = 3) VS<br>ICH-Day 1 (n = 3)<br>ICH-Day 2 (n = 3)<br>ICH-Day 3 (n = 3)	One-way ANOVA	F(3, 8) = 32.7	Sham vs ICH-Day 1: p = 0.8590<br>Sham vs. ICH-Day 2: p = 0.0002<br>Sham vs. ICH-Day 3: p = 0.0004
Figure. S9B	Permeability index	Saline = 0.2922<br>ICH = 1.044<br>ICH+TAT = 1.056<br>ICH+TE1 = 0.9519	Saline (n =4) VS<br>ICH (n = 3)<br>ICH+TAT (n = 3)<br>ICH+te1 (n = 4)	One-way ANOVA	F(3, 10) = 6.9	saline vs ICH: p = 0.0193<br>saline vs ICH+TAT p = 0.0176<br>saline vs ICH+TE1: p = 0.0261<br>ICH vs ICH+TAT: p > 0.9999<br>ICH vs ICH+TE1: p = 0.9685<br>ICH+TAT vs ICH+TE1: p = 0.9554
Figure. S9C	RFU serum	Saline = 48444<br>ICH = 39655<br>ICH+TAT = 39101<br>ICH+TE1 = 40174	Saline (n =4) VS<br>ICH (n = 3)<br>ICH+TAT (n = 3)<br>ICH+te1 (n = 4)	One-way ANOVA	F(3, 10) = 1.848	saline vs ICH: p = 0.3178<br>saline vs ICH+TAT p = 0.2725<br>saline vs ICH+TE1: p = 0.3055<br>ICH vs ICH+TAT: p = 0.9995<br>ICH vs ICH+TE1: p = 0.9995<br>ICH+TAT vs ICH+TE1: p = 0.9958
Figure. S9E	Branch points	Saline = 37.8<br>ICH = 13.75<br>ICH+TAT = 11.3<br>ICH+TE1 = 13	Saline (n =4) VS<br>ICH (n = 4)<br>ICH+TAT (n = 4)<br>ICH+te1 (n = 4)	One-way ANOVA	F(3, 10) = 1.848	saline vs ICH: p < 0.0001<br>saline vs ICH+TAT p < 0.0001<br>saline vs ICH+TE1: p < 0.0001<br>ICH vs ICH+TAT: p = 0.4987<br>ICH vs ICH+TE1: p = 0.9769<br>ICH+TAT vs ICH+TE1: p =0.7635

Supplementary Table 2

Netrin-1（Δ407-443）：
ATGATGCGCGCAGTGTGGGAGGCGCTGGCGGCGCTGGCGGCGGTGGCGTGCCTGG
TGGGCGCGGTGCGCGGCGGGCCCGGGCTCAGCATGTTCGCGGGCCAGGCGGCGCA
GCCCGATCCCTGCTCGGACGAGAACGGCCACCCGCGCCGCTGCATCCCGGACTTTG
TCAATGCGGCCTTCGGCAAGGACGTGCGCGTGTCCAGCACCTGCGGCCGGCCCCC
GGCGCGCTACTGCGTGGTGAGCGAGCGCGGCGAGGAGCGGCTGCGCTCGTGCCAC
CTCTGCAACGCGTCCGACCCCAAGAAGGCGCACCCGCCCGCCTTCCTCACCGACCT
CAACAACCCGCACAACCTGACGTGCTGGCAGTCCGAGAACTACCTGCAGTTCCCG
CACAACGTCACGCTCACACTGTCCCTCGGCAAGAAGTTCGAAGTGACCTACGTGA
GCCTGCAGTTCTGCTCGCCGCGGCCCGAGTCCATGGCCATCTACAAGTCCATGGAC
TACGGGCGCACGTGGGTGCCCTTCCAGTTCTACTCCACGCAGTGCCGCAAGATGTA
CAACCGGCCGCACCGCGCGCCCATCACCAAGCAGAACGAGCAGGAGGCCGTGTG
CACCGACTCGCACACCGACATGCGCCCGCTCTCGGGCGGCCTCATCGCCTTCAGCA
CGCTGGACGGGCGGCCCTCGGCGCACGACTTCGACAACTCGCCCGTGCTGCAGGA
CTGGGTCACGGCCACAGACATCCGCGTGGCCTTCAGCCGCCTGCACACGTTCGGCG
ACGAGAACGAGGACGACTCGGAGCTGGCGCGCGACTCGTACTTCTACGCGGTGTC
CGACCTGCAGGTGGGCGGCCGGTGCAAGTGCAACGGCCACGCGGCCCGCTGCGTG
CGCGACCGCGACGACAGCCTGGTGTGCGACTGCAGGCACAACACGGCCGGCCCGG
AGTGCGACCGCTGCAAGCCCTTCCACTACGACCGGCCCTGGCAGCGCGCCACAGC
CCGCGAAGCCAACGAGTGCGTGGCCTGTAACTGCAACCTGCATGCCCGGCGCTGC
CGCTTCAACATGGAGCTCTACAAGCTTTCGGGGCGCAAGAGCGGAGGTGTCTGCCT
CAACTGTCGCCACAACACCGCCGGCCGCCACTGCCATTACTGCAAGGAGGGCTAC
TACCGCGACATGGGCAAGCCCATCACCCACCGGAAGGCCTGCAAAGCCTGTGATT
GCAGCCGCTCTCCCATCGCCCCCTGCATAAAGATCCCTGTAGCGCCGCCGACGACT
GCAGCCAGCAGCGTGGAGGAGCCTGAAGACTGCGATTCCTACTGCAAGGCCTCCA
AGGGGAAGCTGAAGATTAACATGAAAAAGTACTGCAAGAAGGACTATGCCGTCC
AGATCCACATCCTGAAGGCGGACAAGGCGGGGGACTGGTGGAAGTTCACGGTGAA
CATCATCTCCGTGTATAAGCAGGGCACGAGCCGCATCCGCCGCGGTGACCAGAGC
CTGTGGATCCGCTCGCGGGACATCGCCTGCAAGTGTCCCAAAATCAAGCCCCTCAA
GAAGTACCTGCTGCTGGGCAACGCGGAGGACTCTCCGGACCAGAGCGGCATCGTG
GCCGATAAAAGCAGCCTGGTGATCCAGTGGCGGGACACGTGGGCGCGGCGGCTGC
GCAAGTTCCAGCAGCGTGAGAAGAAGGGCAAGTGCAAGAAGGCCTAG
